# Development of a Well-Characterized Cynomolgus Macaque Model of Marburg Virus Disease for Support of Vaccine and Therapy Development

**DOI:** 10.3390/vaccines10081314

**Published:** 2022-08-14

**Authors:** Kendra J. Alfson, Yenny Goez-Gazi, Michal Gazi, Ying-Liang Chou, Nancy A. Niemuth, Marc E. Mattix, Hilary M. Staples, Benjamin Klaffke, Gloria F. Rodriguez, Carmen Bartley, Anysha Ticer, Elizabeth A. Clemmons, John W. Dutton, Anthony Griffiths, Gabe T. Meister, Daniel C. Sanford, Chris M. Cirimotich, Ricardo Carrion

**Affiliations:** 1 Texas Biomedical Research Institute, 8715 W. Military Dr., San Antonio, TX 78227, USA; 2 Battelle Biomedical Research Center (BBRC), 1425 Plain City Georgesville Road, West Jefferson, OH 43162, USA; 3 Nonclinical Pathology Services, LLC, 5920 Clubhouse Pointe Dr., Medina, OH 44256, USA

**Keywords:** Marburg virus, animal model, animal rule

## Abstract

Marburg virus (MARV) is a filovirus that can infect humans and nonhuman primates (NHPs), causing severe disease and death. Of the filoviruses, Ebola virus (EBOV) has been the primary target for vaccine and therapeutic development. However, MARV has an average case fatality rate of approximately 50%, the infectious dose is low, and there are currently no approved vaccines or therapies targeted at infection with MARV. The purpose of this study was to characterize disease course in cynomolgus macaques intramuscularly exposed to MARV Angola variant. There were several biomarkers that reliably correlated with MARV-induced disease, including: viral load; elevated total clinical scores; temperature changes; elevated ALT, ALP, BA, TBIL, CRP and decreased ALB values; decreased lymphocytes and platelets; and prolonged PTT. A scheduled euthanasia component also provided the opportunity to study the earliest stages of the disease. This study provides evidence for the application of this model to evaluate potential vaccines and therapies against MARV and will be valuable in improving existing models.

## 1. Introduction

*Marburgviruses* are nonsegmented, single-stranded, negative-sense, RNA viruses that belong to the family *Filoviridae.* Marburg virus (MARV) can infect humans and nonhuman primates (NHPs), causing severe disease and death [1,2]. Ebola virus (EBOV) has been the primary filovirus target for research due to recent outbreaks of unprecedented size and in locations where EBOV was not previously endemic, resulting in licensure of vaccines and therapeutics against EBOV [3,4,5]. MARV causes Marburg virus disease (MVD) [6], with an average case fatality rate of approximately 50% [1,2,7,8], and low doses of MARV can be lethal in an NHP model [9]. A natural reservoir for MARV is the Egyptian rousette bat (*Rousettus aegyptiacus*), and there is the potential for spillover events from bats into humans, leading to new outbreaks [10]. Cases of MVD continue to occur, and the west Africa 2013–2016 EBOV outbreak highlighted the risk for filoviruses to cause large, unprecedent outbreaks [11]. There are currently no approved MARV vaccines or therapies. In 2018, the World Health Organization Research and Development Blueprint listed Marburg virus as one of the emerging infectious diseases that should be targeted for research to develop preventative or treatment measures [12].

Characterizing MVD and evaluating vaccines or therapies will necessitate compliance with the US Food and Drug Administration (FDA) Animal Rule (21 CFR 314.60 and 601.90) due to the high mortality rate, sporadic nature of outbreaks, and ethical concerns for conducting traditional efficacy trials [13,14,15]. Approval via this mechanism relies on well-characterized animal model(s) that adequately recapitulate human disease and efficacy endpoints [13,14,15]. However, few models exist for MARV, and a standardized, well-characterized NHP model is still needed [16].

The purpose of this study was to characterize the disease course in cynomolgus macaques exposed to Marburg virus and to identify biomarkers that have the potential to serve as triggers to treat during therapeutic studies under the FDA Animal Rule. Herein, we summarize morbidity and mortality, physiology, clinical pathology, telemetry kinetics, viral load, cytokines/chemokines, and anatomical pathology data collected from cynomolgus macaques exposed intramuscularly (IM) to Marburg virus (*Marburg Marburgvirus*, MARV, Angola variant). Two animals were also mock exposed to sterile phosphate-buffered saline (PBS) as controls.

## 2. Materials and Methods

### 2.1. Ethics Statement

Animal research was conducted under an Institutional Animal Care and Use Committee (IACUC)-approved protocol in compliance with the Animal Welfare Act, Public Health Service (PHS) policy, and federal regulations related to experiments involving animals. Texas Biomedical Research Institute (Texas Biomed) is accredited by AAALAC International. All euthanasia was approved by a veterinarian and was performed using an overdose of sodium pentobarbital. To minimize pain and distress, euthanasia criteria were developed. Briefly, an animal was approved for euthanasia if: the total clinical score was 15 or greater; or the animal scored 8 for responsiveness and also exhibited either a temperature change greater than 5 °F from baseline or changes above a pre-determined threshold in two of the following: γ-glutamyltransferase (GGT), alanine aminotransferase (ALT), alkaline phosphatase (ALP), Albumin (ALB), or blood urea nitrogen (BUN) ([17,18,19]).

### 2.2. Animal Care and Clinical Observations

Ten male and ten female cynomolgus macaques (*Macaca fascicularis,* Chinese origin), 3.39 to 4.10 years of age and weighing between 2.55 and 3.77 kg on the day of exposure were obtained from Envigo (formerly Covance; Alice, TX, USA). Animals were housed individually in standard cages and underwent an acclimation period of seven days at Animal Biosafety Level 4 (ABSL-4), prior to MARV exposure. Animals were seronegative for Simian Immunodeficiency Virus (SIV), Simian T-Lymphotropic Virus-1 (STLV-1), Simian Varicella Virus (SVV), and *Macacine herpesvirus* 1 (Herpes B virus); polymerase chain reaction (PCR) tested negative for Simian Retrovirus (SRV1 and SRV2); PCR and serologically negative for *Trypanosoma cruzi*; negative for tuberculosis; antibody-negative for Ebola Reston (Virus Reference Laboratory, San Antonio, TX, USA); seronegative for Ebola virus, Sudan virus, and Marburg virus glycoprotein (Texas Biomed, San Antonio, TX, USA); showed no signs of active infection with *Salmonella* or *Shigella* bacteria; and were experimentally naive.

Water was available ad libitum, and animals were fed certified primate diet from Purina Mills (Diet 5048). Inanimate enrichment consisted of perches and manipulable toys; food enrichmentwas provided at least five times per week. Excreta pans under the cages, cage flooring, and room floors were cleaned daily.

The targeted environmental conditions were a temperature of 74 °F ± 10 °F and relative humidity of approximately 30 to 70%. The light cycle was approximately 12 h on/12 h off.

Animals were observed at least twice daily by veterinary technicians during the study. Clinical observations involved evaluation of 13 different parameters and assignment of a numerical score to each parameter (previously described [19]). Briefly, the following observations were assigned a score of 1 (for each item, if observed): reduced intake of food or food enrichment or fluid, no stool present, dehydration, appearance (rough hair coat), nasal discharge, bleeding from a blood collection site, ≥10 to <20% body weight loss, 2 to 2.9 °F change in rectal temperature, or mild petechia. The following observations were assigned a score of 2 (for each item, if observed): if the animal is producing a reduced amount of stool, bleeding (from a site other than where blood was collected), not drinking; or exhibiting ≥20% body weight loss, a 3 to 4.9 °F change in rectal temperature, or moderate petechia. A ≥ 5 °F change in rectal temperature or severe petechia each were assigned a score of 3. Labored breathing is assigned in a score of 8 and agonal breathing a score of 15 (which results in immediate euthanasia). Finally, responsiveness is scored as follows: 1 point for slightly diminished general activity, 2 points for reduced response to external stimuli, 8 points for moderate to dramatically reduced response to external stimuli, and 15 points for severe unresponsiveness (which results in immediate euthanasia).

Animals were observed more frequently as clinical signs warranted; a clinical score of 4 to 7 resulted in all animals being observed at least three times per day, and a clinical score greater than 7 resulted in all animals being observed at least four times per day.

### 2.3. Cells and Virus

Vero E6 cells (NR-596; BEI resources, Manassas, VA, USA) were maintained in normal growth media, which consisted of Minimum Essential Media (MEM; Gibco, Grand Island, NY, USA) supplemented with 2 mM of L-glutamine (Gibco, Grand Island, NY, USA), 1 mM of sodium pyruvate (Gibco, Grand Island, NY, USA), and 10% heat-inactivated fetal bovine serum (FBS; Gibco, Grand Island, NY, USA), at 37 °C with 5% CO_2_.

A second-cell culture passage of MARV (Homo sapiens-tc/AGO/2005/Angola-0501379) was supplied by Dr. Tom Ksiazek (at National Institute of Allergies and Infectious Diseases (NIAID’s) World Reference Center for Emerging Viruses and Arboviruses (WRCEVA) at the University of Texas Medical Branch (UTMB Health) Galveston National Laboratory) in 2012. The exposure stock virus was propagated from this via passaging one time on Vero E6 cells at a multiplicity of infection of 0.001 to generate a passage 3 virus stock [9]. The stock was characterized to assess titer, purity, and microbial or toxin contamination, and was confirmed to be wild-type Marburg virus (Angola variant) by deep sequencing (GenBank accession#: KR063674.1; sequence provided to GenBank by J. Craig Venter Institute).

Sterile PBS (lot number 1967646) was used to mock infect the control animals. Sterile PBS was stored at room temperature.

### 2.4. Marburg Virus Exposure

All animals were housed in the ABSL-4 laboratory; eighteen were exposed to Marburg virus, and two were mock exposed to sterile PBS. Animals were randomly assigned to groups to ensure veterinary staff remained blinded to the exposure material.

Prior to exposure, NHPs were sedated via IM injection with Telazol (Zoetis Inc., Parsippany-Troy Hills, NJ, USA), and each animal was exposed in the right deltoid muscle of the arm to 0.5 mL of MARV Angola or sterile PBS via IM injection.

After exposure, animals were sedated on scheduled time points (Days −7, 0, 3, 5, 7, 9, 11, 13, and 14, relative to exposure) for monitoring of rectal temperatures and body weight, and for blood collection to assess clinical pathology and viremia. Sedation and blood collection were also performed prior to all scheduled and unscheduled euthanasia. Ten virus-exposed animals were scheduled for euthanasia on pre-determined days post exposure (two animals scheduled per day): 2, 3, 5, 7, and 9; one animal scheduled for euthanasia on Day 9 was found dead on Day 9, prior to euthanasia. The mock-exposure control animals were euthanized as scheduled on Day 14 post exposure.

### 2.5. Verification of Exposure Dose

For the 1000 PFU exposure, the MARV Angola exposure stock (titer 4.4 × 10^6^ PFU/mL) was diluted to a target concentration of 2000 PFU/mL in sterile PBS. Mock-exposed animals received undiluted, sterile PBS. The target dose of 1000 PFU was chosen to ensure lethality, based on convention in the filovirus field, and for consistency with other studies. However, lower doses are sufficient to cause full lethality [9]. Following preparation and prior to injection into animals, an aliquot was removed for verification of infectivity via a neutral red agarose overlay (NRAO) plaque assay [19,20], and the average titer was 444 PFU/mL (standard deviation = 252 PFU/mL). The remaining exposure material after administration to the final animal was also evaluated in the NRAO plaque assay, and the titer was 721 PFU/mL (standard deviation = 359 PFU/mL). The difference between pre-exposure and post-exposure values was not surprising and was within the expected range of variability for the plaque assay (≤0.5 log10).

### 2.6. Determination of Infectious Viral Titers and Viral RNA

Viremia was determined via plaque assays performed on serum collected on Days 0, 3, 5, 7, 9, 11, 13, and 14 post exposure, and collected prior to any euthanasia. During necropsy, samples of tissues were also aseptically collected from each animal for determination of viral load. Plaque assays were performed by the NRAO method [19,20].

Viral RNA was quantified from serum and tissue samples via quantitative reverse transcription-PCR (qRT-PCR) using an Applied Biosystems Quant Studio 3 real-time PCR instrument (Life Technologies Corporation, Carlsbad, CA, USA). One-step qRT-PCR was performed using RNA UltraSense One-Step Quantitative RT-PCR System (Invitrogen, Carlsbad, CA, USA) and primers and probe specifically designed to detect a region of the MARV glycoprotein (GP) gene; this assay will detect all RNA corresponding to the MARV virus GP gene, including antigenome and mRNA. The primer and probe sequences were as follows: *Marburg marburgvirus* Forward Primer: 5′ GGC CTT CAG GGC AGG TGT A 3′; *Marburg marburgvirus* Reverse Primer: 5′ CCT GTG CAT GAG GGT TTT GA 3′; *Marburg marburgvirus* Probe: 6-FAM 5′ CCT TGC TGT TAG ATC CTC CTA CCA A 3′.

Mock-exposed animals exhibited no detectable levels of infectious virus and no detectable viral RNA in the serum. Both exhibited detectable viral RNA in samples collected from the adrenal gland, and animal 590 exhibited low levels in samples collected from the spleen, heart, and challenge site. The results bordered on the detection limit of the qRT-PCR and did not seem to result from genuine amplification. Thus, for each of these tissues (adrenal gland, spleen, heart, and challenge site), the highest value exhibited by the mock-exposed animals was used as the cut off, and any exposed animals with values below that level were also labeled below detection limit. This impacted two spleen samples from animals euthanized on Day 2 and Day 3.

### 2.7. Telemetry to Measure Body Temperature and Activity

Animals were implanted with Data Sciences International (DSI, New Brighton, MN, USA) M00 telemeters to measure body temperature and activity. Upon study completion, hourly averages were compared to the baseline established before exposure. Data were omitted in cases of signal drop out or non-physiological values.

### 2.8. Coagulation, Complete Blood Counts, and Clinical Chemistry

Blood was collected at scheduled time points (Days −7, 0, 3, 5, 7, 9, 11, 13, and 14, relative to exposure). Coagulation times were determined on whole blood collected with no additives, using an IDEXX Coag Dx Analyzer (IDEXX Laboratories, Westbrook, ME, USA). These blood samples were processed to obtain the serum. Serum was analyzed to measure C-reactive protein (CRP) levels using a Piccolo BioChemistry Panel Plus on a Vet Scan analyzer (Abaxis, Inc., Union City, CA, USA). Whole blood was also collected into tubes containing ethylenediaminetetraacetic acid (EDTA) for complete blood counts (CBC) using a Procyte Dx Hematology Analyzer (IDEXX laboratories, Westbrook, ME, USA) and for clinical chemistry using the mammalian liver enzyme profile rotor on a Vet Scan analyzer (Abaxis, Inc., Union City, CA, USA).

### 2.9. Cytokines and Chemokines Analysis

The MILLIPLEX MAP Non-Human Primate Cytokine Magnetic Bead Panel: Immunology Multiplex Assay (PRCYTOMAG-40K-22C, Millipore Corporation, Billerica, MA, USA) was used to measure serum levels of the following cytokines and chemokines: granulocyte colony-stimulating factor (G-CSF), granulocyte-macrophage colony-stimulating factor (GM-CSF), interferon gamma (IFN-γ), interleukin (IL)-1ra, IL-1β, IL-2, IL-4, IL-5, IL-6, IL-8, IL-10, IL-12/23 (p40), IL-13, IL-15, IL-17, IL-18, monocyte chemotactic protein-1 (MCP-1), macrophage inflammatory protein (MIP)-1α, MIP-1β, soluble CD40 ligand (sCD40L), transforming growth factor alpha (TGF-α), vascular endothelial growth factor (VEGF) [19].

### 2.10. Necropsy and Pathology

All animals were necropsied to examine all external surfaces, all orifices, and the cranial, thoracic, and abdominal cavities and their contents. The following tissues were collected for viral load and histopathology analysis: skin and underlying subcutis and muscle at the exposure site; the right inguinal, right axillary, mediastinal, and mesenteric lymph nodes; gastrointestinal tract tissues, including stomach, duodenum, jejunum, ileum with gut-associated lymphoid tissue (GALT), colon, and rectum; heart, spleen, liver, adrenal gland, kidney, and brain. The right upper lobe of the lung was collected for histopathology, and the lower left lobe of the lung was collected for viral load analysis. Samples were aseptically removed and divided into a dry vial for freezing or a cassette for fixation in 10% neutral-buffered formalin for histopathologic examination. Following fixation, tissue samples were embedded in paraffin, sectioned and stained with hematoxylin and eosin, and evaluated microscopically by a board-certified veterinary pathologist.

### 2.11. Blinding and Randomization

Animals were randomly assigned to an exposure group and to scheduled euthanasia time points using Stata (version 12.1; StataCorp. 2011. Stata Statistical Software: Release 12. College Station, TX: StataCorp LP); random seed values were selected using R (version 2.14.1; R Core Team. R Foundation for Statistical Computing, Vienna, Austria). The following staff were blinded to the identity of the mock-exposed animals: veterinary technicians and veterinarians until finalization of post-in life analysis, in vitro staff performing viral load determination and biomarker analysis, individuals performing necropsy, the board-certified veterinary pathologist during microscopic evaluation of stained slides (this individual was unblinded for preparation of the histopathology report).

### 2.12. Statistics

Statistical analyses were performed if at least 2 animals per group were available for analysis. Analysis of variance (ANOVA) models were fitted to data with effects for group, study time point, and the interaction between group and study time point to assess the model assumption of normality and to identify potential outliers. Different ANOVA models were fitted to the change from baseline data separately at each time point to determine if there were significant changes from the pre-challenge baseline within a group or differences between the groups. Statistical analyses were conducted using SAS^®^ (version 9.4; SAS, Cary, NC, USA) on the 64-bit platform. Results are reported at the 0.05 level of significance.

### 2.13. Quality Standards

To ensure an adequate and well-controlled study, the study adhered to a study protocol, a quality agreement that was consistent with the principles of Good Laboratory Practices (GLP), applicable Texas Biomed Standard Operating Procedures (SOPs), and generally recognized good documentation practices. A study director, the primary point of control, was responsible for the conduct of work and reporting of data. Any needed changes to the approved protocol were documented via study protocol amendments, and deviations from SOPs or the study protocol were documented and immediately reported to the study director.

## 3. Results

### 3.1. Experimental Design

Animals were exposed to a target dose of 1000 plaque-forming units (PFU) of MARV (n = 18) or mock exposed to sterile phosphate-buffered saline (PBS) (n = 2). Ten (10) MARV-exposed animals were part of a planned serial euthanasia in order to characterize pathology from multiple time points post exposure. To confirm that the exposure dose for each iteration was lethal and to measure physiology, clinical pathology, and telemetry kinetics, eight MARV-exposed animals were not scheduled for euthanasia. Many biological parameters, including body weight and rectal body temperature changes and clinical pathology, were monitored. Viremia and cytokines/chemokines were also measured.

### 3.2. Clinical Progression of Disease

#### 3.2.1. Mortality

**Figure 1:**
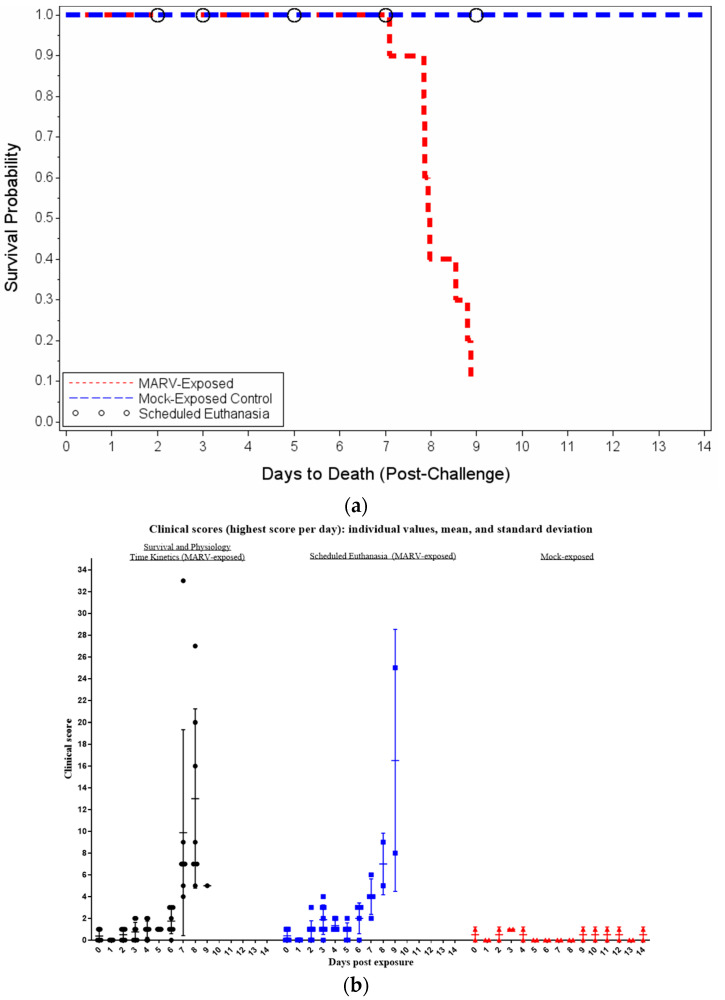
Survival and Clinical Scores. (**a**) Kaplan–Meier Time to Death Plot. (**b**) Daily Clinical Scores (Highest Score Per Day) in Cynomolgus macaques exposed to MARV or mock exposed: Group comparison with individual values, mean, and standard deviation displayed. One animal in the scheduled euthanasia group was found dead on the day of the scheduled euthanasia; this animal is included in the scheduled euthanasia group in the figure.

The two control animals remained healthy throughout and were euthanized as scheduled on Day 14 post exposure. Within the scheduled euthanasia group, two animals were euthanized as planned on Days 2, 3, 5, and 7. One animal in the scheduled euthanasia group for Day 9 was found dead on the day of the scheduled euthanasia, and the other animal was euthanized as planned on Day 9. Within the MARV exposure group not scheduled for euthanasia, four animals were humanely euthanized due to moribundity: one on Day 7 post exposure and three on Day 8 post exposure. Four animals were found dead: two on Day 8 post exposure and two on Day 9 post exposure. Mortality is summarized in Table 1. Kaplan–Meier median time to death was 7.95 days for the MARV-exposed group (Figure 1). Animals that were scheduled for euthanasia on Days 2, 3, 5, 7, or 9 were included in the analysis and censored at the scheduled euthanasia time.

#### 3.2.2. Clinical Scores

Clinical observations for each animal involved the evaluation of thirteen different parameters and assignment of a numerical score to each parameter [19]. These parameters included: feed (and enrichment) and fluid intake (and dehydration), stool output, bleeding or nasal discharge, rectal temperature, body weight, appearance, petechia, breathing, and responsiveness Scores were then added to achieve a total clinical score for each observation period. Figure 1 displays the individual values, mean, and standard deviation of the highest clinical scores for MARV-exposed animals versus mock-exposed animals. Clinical scores of 0 and 1 were observed during the pre-exposure baseline period in the MARV and mock-exposed groups; occasional scores of 1 were due to reduced feed consumption or stool output. Clinical scores did not exceed 1 for either of the mock-exposed control group animals throughout the study. On the day of exposure, eight animals scored 1 for reduced feed consumption.

For MARV-exposed animals, few animals exhibited clinical signs that warranted scores above 0 prior to Day 3; ten scored 1 for reduced feed consumption, and one scored 3 on Day 2 for diarrhea and reduced feed consumption. Animals 594 and 597 were euthanized on schedule on Day 2 with clinical scores of zero. Over Days 3 to 4, most animals exhibited low scores, with the highest score being 4, for reduced consumption or abnormal stool output or temperature changes. Animals 585 and 605 were euthanized on schedule on Day 3, with scores of 4 and 1, respectively.

By Days 5 and 6, four animals exhibited rectal body temperature increases; fourteen also scored for reduced feed consumption; five of these also presented with reduced or no stool output, and one also presented with reduced fluid input. Animals 592 and 602 were euthanized on schedule on post-exposure Day 5, with scores of 1 (for reduced feed consumption) and 2 (for rectal body temperature change and reduced feed consumption), respectively.

On Day 7, more severe symptoms appeared in the MARV-exposed group. Eight animals exhibited slightly diminished activity or slightly reduced responses to external stimuli. All animals except 584 exhibited reduced intake in feed and/or fluid, and/or reduced (or lack of) stool output. Bleeding manifestations first appeared in seven animals; six presented with petechial rash (mild to severe degree), and one was found bleeding from a site other than the blood collection site. On Day 7, animals 589 and 603 were euthanized on schedule, both with clinical scores of 4. Animal 591 was euthanized due to moribundity with a score of 33.

Scores remained high on Day 8, with all MARV-exposed animals scoring for reduced intake in feed and/or enrichment and/or fluid, and/or reduced (or lack of) stool output, and/or dehydration, slightly diminished activity or slightly reduced responses to external stimuli, or prostration, unless stimulated. Five animals succumbed on Day 8 post exposure: animals 588, 593, and 596 were euthanized after reaching total clinical scores between 16 and 27, and animals 601 and 604 were FDIC. These five animals exhibited clinical scores for decreased rectal body temperatures (up to 16.8 °F decrease from baseline). Animals 587 and 600 succumbed to disease the following day.

#### 3.2.3. Temperature, Activity, and Body Weight

Rectal temperatures ranged from 98.1 °F to 103.4 °F (average 100.3 °F) for mock-exposed animals throughout the study and for MARV-exposed animals pre-exposure (from Days −7 to 0). Baseline was calculated as an average of the animal’s rectal temperature on Day −7 and Day 0; all changes discussed herein are in relation to baseline.

Temperature changes from baseline were minimal (<1.3 °F) until Day 5, when four animals (591, 595, 601, and 602 (scheduled euthanasia)) exhibited rectal body temperature increases that warranted clinical scores (2.0 °F to 2.5 °F). On Day 7, five animals (584, 588, 600, 603, and 604) had rectal temperatures above baseline that warranted clinical scores (2.5 °F to 4.7 °F).

At the time of death on Day 7, animal 603 had a rectal body temperature of 103.7 °F (increased 3.5 °F relative to baseline). At the time of death on Day 8, three animals that were humanely euthanized (588, 593, and 596) exhibited decreased rectal temperatures (11.3 °F, 4.1 °F, and 12.1 °F, respectively). On Day 9, animal 584 (scheduled euthanasia) exhibited a decrease of 13.2 °F (relative to baseline) in rectal body temperature.

The mean increase from Day 0 rectal temperature was statistically significant for MARV-exposed animals on Study Days 5 and 7. There was a significant group effect on Study Day 5.

**Figure 2:**
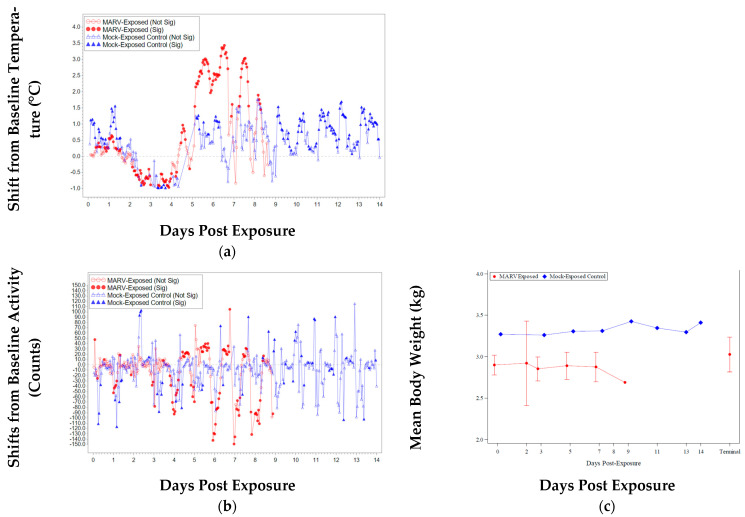
Temperature, Activity, and Weight. Terminal—Terminal data collected from animals that met euthanasia criteria (unscheduled euthanasia) or were found dead were combined and reported as a single time point. (**a**) Least-Square Means Plot for Shift from Baseline Body Temperature (°C) from telemetry. (**b**) Least-Square Means Plot for Shift from Baseline Activity (Counts). (**c**) Plot of Mean Body Weight (kg) with 95% Confidence Interval Over Time (Confidence interval was not plotted for sample size less than 3).

Animals were also implanted with M00 telemeter implants (DSI) to measure temperature and activity (Figure 2a,b, respectively). Telemetry data were unavailable for animal 596, and no temperature/activity telemetry data were available for the entire study cohort between Day −1 relative to exposure and the morning of exposure. Fever onset was defined as the first time point when an animal’s mean temperature value was greater than 39 °C. Mock-exposed animals did not exhibit fever during the study; peak temperature for these animals ranged from 37.8 to 38.2 °C (average 38.0 ± 0.3 °C). Animals subject to scheduled euthanasia on Days 2 and 3 (n = 4) and animal 592 (scheduled euthanasia on Day 5) did not exhibit fever.

Twelve MARV-exposed animals exhibited fever: 67% (n = 8) on Day 4, 25% (n = 3) on Day 5, and animal 584 on Day 6. Peak temperature for these animals ranged from 39.7 to 41.0 °C (average 40.5 °C ± 0.4; median 40.6 °C). The time of peak temperature ranged from Day 5 to Day 7, with unscheduled euthanasia animals that succumbed to exposure experiencing their peak temperature 2 to 3 days prior to euthanasia or being found dead.

Mean activity data for the animals illustrated the expected diurnal pattern for all animals until approximately Day 5 post exposure (Figure 2b). Mock-exposed animals exhibited a consistent activity pattern throughout the study, while MARV-exposed animals exhibited erratic mean activity as other clinical signs progressed.

For body weight, the mean decrease from baseline (Study Day 0) was statistically significant for animals in the MARV-exposed group on Study Days 3 and 7. There was a significant group effect on Study Day 7 (*p* < 0.05). Figure 2c displays the group mean changes from baseline weight.

#### 3.2.4. Clinical Pathology (Complete Blood Counts, Clinical Chemistry, and Coagulation)

Complete blood counts and clinical chemistry analyses were performed on whole blood collected from MARV-exposed and mock-exposed animals at scheduled time points during the study. Various hematology parameters were significantly increased or decreased from baseline on at least one study day in each of the MARV-exposed and mock-exposed groups. Significant group effects were also noted for at least one study day for different populations of white blood cells (WBCs): absolute counts and relative percentages for monocytes, eosinophils, and basophils; and absolute count for lymphocytes.

**Figure 3:**
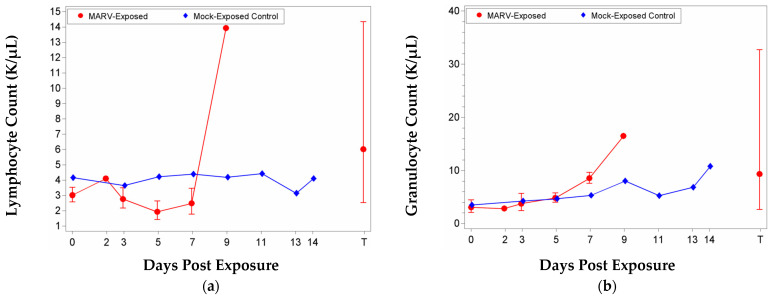

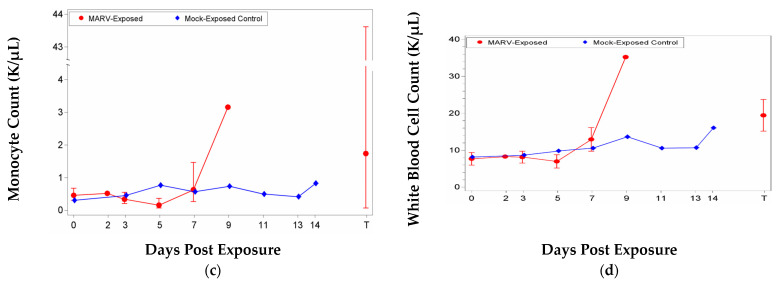
Mean with 95 percent confidence intervals over the course of the study for white blood cell parameters. Confidence interval was not plotted for sample size less than 3. Unless otherwise noted, values were log-transformed, and the geometric mean is displayed. Terminal (T)—Terminal data collected from animals that met euthanasia criteria (unscheduled euthanasia) were combined and reported as a single time point. (**a**) Lymphocyte counts; (**b**) Granulocyte counts; (**c**) Monocyte counts; (**d**) White blood cell counts—values for this parameter were not log-transformed; arithmetic mean displayed. For (**a**–**c**), the 95 percent confidence intervals are not symmetric because geometric confidence bounds are displayed on a linear scale.

For MARV-exposed animals, on Day 3, there were significant decreases from baseline in lymphocyte relative percentages and a corresponding increase in relative percentages of granulocytes. This continued, and on Day 5, there was a significant decrease from baseline for MARV-exposed animals for lymphocytes and also monocytes (absolute counts and relative percentages) and a corresponding increase in granulocytes (absolute counts and relative percentages); the group effect was significant for monocytes. By Day 7, relative percentages for lymphocytes remained decreased, and there were significantly increased total WBC (absolute counts) and granulocytes (absolute counts and percentages). On Day 9, there was a significant increase as a proportion of baseline in lymphocyte absolute counts; the group effect was also significant on this day. Figure 3 displays mean absolute counts over time for each group.

For both eosinophils and basophils (absolute counts and relative percentages), the group effects were significant on Days 3 and 5; eosinophils absolute counts also showed group effects on Day 9. However, on Days 3 and 5, these effects were not associated with a clear increase or decrease from baseline. For eosinophils (absolute counts and relative percentages), there were significant increases from baseline for both groups on Study Day 9. For the mock-exposed group, there were significant decreases from baseline in absolute counts and relative percentages on Days 3 and 5, and significant increases in relative percentages on Days 11 and 13. For basophils, when potential outliers were excluded, there was a significant decrease as a proportion of baseline for the mock-exposed group on Study Day 3 (percent and count) and a significant increase as a proportion of baseline for the MARV-exposed group on Study Day 5 (count).

**Figure 4:**
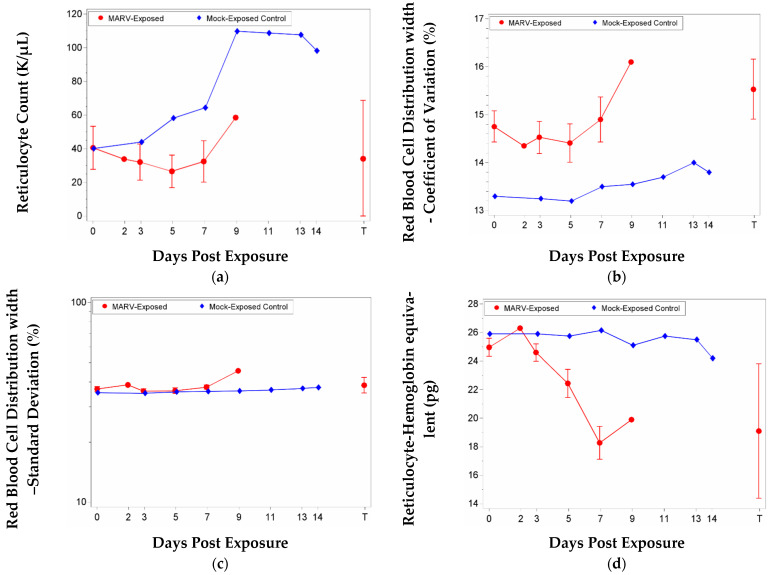
Mean with 95 percent confidence intervals for red-blood-cell-related parameters. Confidence interval was not plotted for sample size less than 3. Unless otherwise noted, values were not log-transformed; arithmetic mean displayed. Terminal (T)—Terminal data collected from animals that met euthanasia criteria (unscheduled euthanasia) were combined and reported as a single time point. (**a**) Reticulocyte counts; (**b**) Red Blood Cell Distribution width—Coefficient of Variation; (**c**) Red Blood Cell Distribution width—Standard Deviation (RDW-SD); values for this parameter were log-transformed, and the geometric mean is displayed; (**d**) Reticulocyte hemoglobin equivalent.

Changes in certain parameters related to red blood cells (RBC) can be symptomatic of several conditions, including anemia and bleeding. However, within this study, decreases in RBCs (along with concomitant increases in reticulocytes), hematocrit (HCT), hemoglobin (HGB), may have been related to repeated blood collection, as there were significant decreases from baseline for both groups on Study Day 7, and there was no significant group effect on any study day. Reticulocyte counts and percentages exhibited a significant decrease from baseline for the MARV-exposed group on Study Day 3; the increase seen in mock-exposed animals may be due to repeated blood collections. Red blood cell distribution width coefficient of variation (RDW-CV) and red blood cell distribution width standard deviation (RDW-SD) also exhibited significant decreases from baseline on Study Day 3 and a significant increase from baseline on Study Day 9. The group effect was significant on Study Day 9. Reticulocyte hemoglobin equivalent (RET-He) exhibited significant decreases from baseline on Study Days 5, 7, and at Terminal. The group effect was significant on Study Day 7. Data are shown in Figure 4.

**Figure 5:**
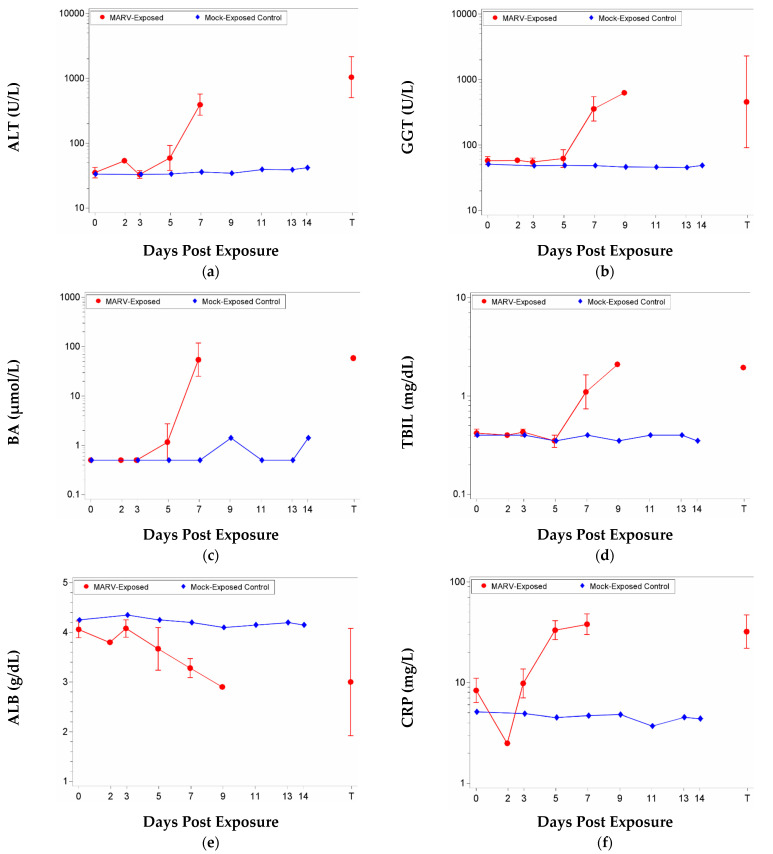
Mean with 95 percent confidence intervals over the course of the study for clinical chemistry parameters. Confidence interval was not plotted for sample size less than 3. Unless otherwise noted, values were log-transformed, and the geometric mean is displayed. Terminal (T)—Terminal data collected from animals that met euthanasia criteria (unscheduled euthanasia) were combined and reported as a single time point. On Day 9, Animal 584 values for ALT, BA, and CRP were not able to be detected (likely as a result of being too high, out of range) and thus are not graphed. (**a**) ALT; (**b**) GGT; (**c**) BA; (**d**) TBIL; (**e**) ALB—this parameter was not log-transformed, arithmetic mean displayed; (**f**) CRP.

Clinical chemistry analyses were performed on whole blood (collected in tubes containing EDTA) collected from MARV-exposed and mock-exposed animals at the scheduled time points during the study and prior to euthanasia. Mock-exposed animals did not exhibit significant changes in clinical chemistry parameters. MARV-exposed animals euthanized on or after Day 7 showed changes in clinical chemistry parameters (ALT, GGT, BA, TBIL, and ALB) indicative of marked liver malfunction (Figure 5a–e). C-reactive protein (analysis performed on serum), a marker of inflammation, was also increased (Figure 5f).

For ALT and CRP, there were significant increases as a proportion of baseline for the MARV-exposed group on Days 5 and 7 and at terminal collection. The group effect was significant for both on Day 7, and for CRP, also on Day 5. For GGT, there were significant increases as a proportion of baseline for the MARV-exposed group on Days 7 and 9 and at terminal collection. The group effects were significant on Days 7 and 9. There was a significant increase as a proportion of baseline on Day 7 for TBIL and BA. For BA, the group effect was significant on Day 7, and for TBIL, when potential outliers were excluded, there was a significant group effect on Day 7. In addition, ALB exhibited significant decreases from baseline for the MARV-exposed group on Days 7 and 9 and at terminal collection. The group effects were significant on Days 7 and 9. When potential outliers were excluded, there was also a significant decrease in ALB from baseline for the MARV-exposed group on Day 5.

For ALP, there was a significant decrease as a proportion of baseline for the MARV-exposed group on Day 3. For BUN, there was a significant decrease as a proportion of baseline for the MARV-exposed group on Day 5. GGT also exhibited significant decreases as a proportion of baseline for the MARV-exposed group on Day 3.

**Figure 6:**
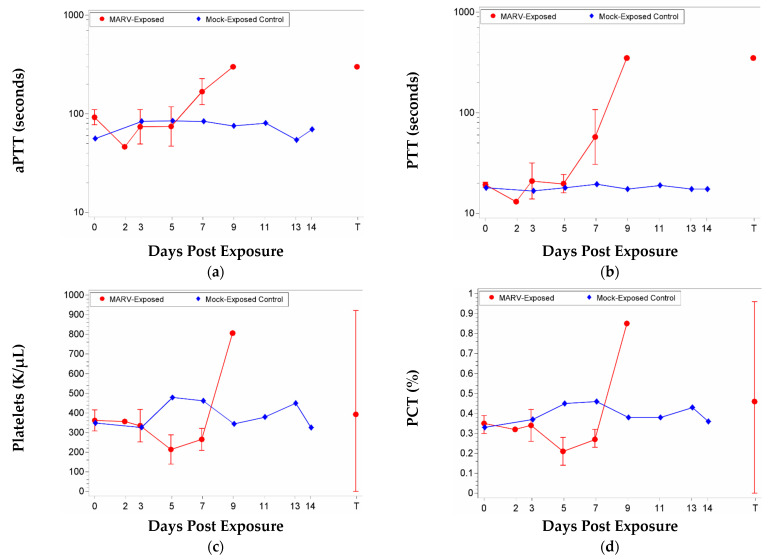
Mean with 95 percent confidence intervals over the course of the study for coagulation. Confidence interval was not plotted for sample size less than 3. For aPTT and PTT, values were log-transformed, and the geometric mean is displayed. For platelets and plateletcrit, values were not log-transformed, and the arithmetic mean is displayed. Terminal (T)—Terminal data collected from animals that met euthanasia criteria (unscheduled euthanasia) were combined and reported as a single time point. (**a**) aPTT; (**b**) PTT; (**c**) Platelets; (**d**) Plateletcrit.

Coagulation was measured by analyzing the activated partial thromboplastin time (aPTT), prothrombin time (PTT), and platelet count at the scheduled time points post exposure and prior to euthanasia (Figure 6). The data indicate coagulopathy evidenced by decreased platelets beginning on Day 5 and prolonged clotting times beginning on Day 7. The MARV-exposed group experienced significant increases from baseline on Study Day 7 and at Terminal for aPTT, and on Study Days 7 and 9 for PTT. The group effect was significant on Study Day 9 for PTT. Platelet counts and plateletcrit (PCT) exhibited significant decreases from baseline for the MARV-exposed group on Study Days 5 and 7. The group effect was significant on Study Day 5.

#### 3.2.5. Cytokine and Chemokine Expression

Serum collected at the scheduled time points and prior to euthanasia was analyzed for the profiles of specific cytokines and chemokines using the MILLIPLEX MAP Non-Human Primate Cytokine Magnetic Bead Panel. The analytes assayed included the following: granulocyte colony-stimulating factor (G-CSF), granulocyte-macrophage colony-stimulating factor (GM-CSF), interferon gamma (IFN-γ), interleukin (IL)-1ra, IL-1β, IL-2, IL-4, IL-5, IL-6, IL-8, IL-10, IL-12/23 (p40), IL-13, IL-15, IL-17, IL-18, monocyte chemotactic protein-1 (MCP-1), macrophage inflammatory protein (MIP)-1α, MIP-1β, soluble CD40 ligand (sCD40L), transforming growth factor alpha (TGF-α), vascular endothelial growth factor (VEGF).

**Figure 7:**
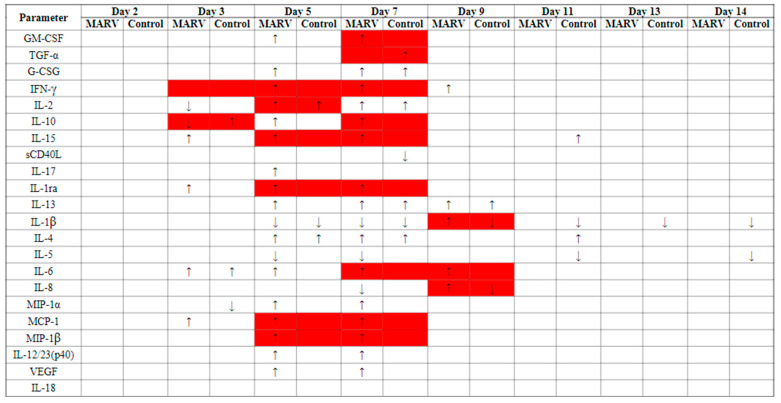
Cytokine and chemokine changes from baseline throughout study for MARV-exposed versus mock-exposed group. Arrows (↑, ↓) indicate that the mean on the study day was significantly greater (“↑”) or less (“↓”) than that at the baseline (Day 0) at the 0.05 level. Red shaded cells indicate that the change from baseline for the MARV-exposed group was significantly different to that of the mock-exposed control group at the 0.05 level. For days 11, 12, and 14, only samples from mock-exposed animals were available.

Filovirus infections in humans and NHPs often result in the release of many cytokines and chemokines, many of which are involved in inflammation and endothelial permeability and likely originate from monocytes and macrophages [21,22,23,24,25]. In this study, many of these parameters exhibited significant (*p* < 0.05) increases from baseline (Figure 7, Supplementary Figure S1). Some of these cytokines and chemokines were increased as early as Day 3 (IL-6, MCP-1, IL-1Ra, and IL-15), with the majority being increased on Days 5 through 9.

Many interleukins that are elevated during human disease and in NHP models were elevated throughout the study [21,22,23,25]. For example, increased IL-6 has frequently been observed in humans and NHPs infected with filoviruses. It is postulated that IL-6 may play a role in the coagulopathy seen during Ebola virus disease (EVD) and MVD and in viral dissemination via recruitment of monocytes [21]. In MARV-exposed animals, there were significant increases as a proportion of baseline throughout the study (Days 3, 5, 7, and 9); group effects were significant on Days 7 and 9. Similarly, IL-1β is another commonly increased cytokine that is likely involved in endothelial cell permeability, which can be a main factor in disease [21]. In MARV-exposed animals, there were significant decreases from baseline on Days 5 and 7 and a significant increase from baseline on Day 9, and there was a significant group effect on Day 9. In addition, in MARV-exposed animals, there were significant increases from baseline for: IL-15 on Days 3, 5, and 7 (the group effect was significant on Days 5 and 7); IL-2 on Days 5 and 7 (the group effect was significant on Day 5); IL-10 on Days 5 and 7 (the group effect was significant on Days 3 and 7); and IL-8 on Day 9 (the group effect was significant on Day 9). Recent research has also shown that during fatal filovirus infections, there are high levels of IL-1Ra [26]. For MARV-exposed animals, there were significant increases on Days 3, 5, and 7; group effects were significant on Days 5 and 7.

While data from human infections are sparse, MARV-exposed NHPs exhibit increased chemoattractants MCP-1, MIP-1α, and MIP-1β [21,25]. For the MARV-exposed animals, there were significant increases as a proportion of baseline for MIP-1α and MIP-1β on Days 5 and 7 (MIP-1β showed significant group effects on Days 5 and 7), and MCP-1 on Days 3, 5, and 7 (significant group effects on Days 5 and 7).

Filoviruses inhibit IFNγ-induced gene expression through various mechanisms. While elevated IFNγ is observed during filovirus infections [21,22,23,25], it is still unclear how it functions during disease. Increased levels are seen during fatal cases and perhaps lead to T-cell apoptosis or endothelial permeability; however, IFNγ may be beneficial for viral clearance and host protection [21]. In MARV-exposed animals, IFNγ exhibited significant increases on Days 5, 7, and 9; group effects were significant on Days 3, 5, and 7.

### 3.3. Virologic Progression of MARV Disease

#### 3.3.1. Viremia

The concentrations of MARV genome equivalents and infectious MARV in the serum collected at scheduled time points and prior to euthanasia were measured by qRT-PCR and plaque assay, respectively.

For MARV-exposed animals, the levels of MARV RNA and infectious MARV increased over time until Day 9 (Table 2, Supplementary Figure S2 and Table S1). One animal euthanized on Day 2 (animal 597) exhibited detectable levels of infectious virus in the serum taken during terminal collection. Ten out of fifteen MARV-exposed animals (67%, data for one animal were undetermined) had detectable levels of infectious virus in the serum on Day 3 post exposure. On Days 5 and 7, all remaining MARV-exposed animals for which sample was available exhibited detectable levels of infectious virus in the serum (animal 584 did not have sample available for analysis on Day 5), ranging from 1.69 × 10^6^ to 3.00 × 10^8^ PFU/mL on Day 7. The three available terminal samples (animals 588 and 593 on Day 8 and animal 584 on Day 9) had more than seven logs of infectious virus per mL of serum. Viral RNA data are displayed in Supplementary Table S1.

#### 3.3.2. Tissue Burden

The concentrations of MARV genome equivalents and infectious MARV in tissues collected at necropsy were measured by qRT-PCR and plaque assay, respectively. More than half of the MARV-exposed animals had detectable MARV RNA and infectious MARV in all tissues, except for the lung and gastrointestinal tract.

Infectious virus was detected starting on Day 2 in the following tissues: spleen, right axillary lymph node, and lung. For animals euthanized on Day 3, animal 605 exhibited detectable levels of infectious virus in the spleen, liver, right axillary lymph node, and adrenal gland. Animal 585 had infectious virus in the liver and the exposure site.

Infectious virus was detected in the small intestine and/or rectum and/or colon after Day 3. All lymph node samples were positive for infectious virus after Day 5. After Day 5, the virus was detected in all of the tissues tested, with the exception of the duodenum from animal 588 (<900 PFU/g on Day 8, data not available from testing with a lower dilution series). Infectious viral load data are shown in Table 3, Table 4 and Table 5 and select tissues in Supplementary Figure S2. Viral RNA data are displayed in Supplementary Tables S2 and S3.

### 3.4. Pathologic Progression of SUDV Disease

No significant macroscopic observations were noted in the two mock-exposed animals at the Day 14 post-exposure terminal necropsy; animal 598 exhibited an enlarged inguinal lymph node that had no correlation with a microscopic lesion. Animals that were scheduled for euthanasia on Days 2 and 3 also had no significant lesions and no significant microscopic changes related to MARV infection. Animal 602 euthanized on Day 5 also presented no significant macroscopic lesions at necropsy, and both animals euthanized on Day 5 exhibited minimal microscopic changes.

Animals exposed to MARV with scheduled euthanasia on Days 5 to 9 presented with some common macroscopic observations: discoloration attributable to rash in the skin (e.g., limbs, axillary and inguinal area, face) (three out of five); enlargement and discoloration of the right axillary lymph nodes (two out of five); discoloration of the lungs (one out of five); friability of the spleen (two out of five) and/or liver (three out of five); and soft contents in colon (one out of five); animal 595 is excluded from these numbers due to being FDIC rather than undergoing scheduled euthanasia as planned on Day 9.

The MARV-exposed group of animals that were euthanized due to moribundity or were found dead on Days 7 to 9 post exposure also had some common macroscopic observations: discoloration attributable to rash on the skin (nine out of nine) or the challenge site (four out of nine); enlargement (eight out of nine), discoloration (seven out of nine), and/or firmness of the lymph nodes (three out of nine); enlargement (six out of nine) or discoloration (one out of nine) of the spleen; discoloration (nine out of nine) and/or friability (seven out of nine) of the liver; discoloration of the urinary bladder mucosa (two out of nine), and discoloration of the testicular parenchyma (two out of five). Some other sporadic findings included the presence of red fluid in the abdominal cavity (animal 593), thickness of the jejunum/duodenum (animal 587), discoloration of the serosa and mucosa of the colon (animal 600), and discoloration of the adrenal glands (animal 601).

The earliest histopathologic change attributable to MARV infection was sinus histiocytosis (noted as early as Day 3) in the lymph nodes. Histopathologic changes associated with fatal MARV infection were present in MARV-exposed animals as early as Day 5 scheduled euthanasia, including: inflammation at the challenge site; lymphoid depletion and lymphocytolysis of the lymph nodes accompanied by fibrin deposition, hemorrhage, and necrosis; lymphoid depletion with lymphocytolysis and fibrin deposition within the spleen; hepatocellular single cell necrosis and inflammation within the liver and necrosis within the cortex of the adrenal glands. Half of the animals that died on Day 9 exhibited paracortical hyperplasia in the lymph nodes, characterized by large immunoblasts within the paracortex. Representative images of tissues most affected histologically (axillary lymph node, spleen, and liver) are included in Supplementary Figures S3–S5.

## 4. Discussion

The purpose of this study was to characterize disease course in cynomolgus macaques intramuscularly exposed to a target dose of 1000 PFU of MARV Angola variant (n = 18). Two mock-exposed control animals were also included for comparison. Body weight, body temperature, telemetry kinetics, clinical pathology, and viremia were monitored from data collected at multiple time points post exposure. One group (n = 10) of MARV-exposed animals were scheduled for serial euthanasia in order to characterize the pathology and tissue viral load from multiple time points.

The two mock-exposure control animals did not exhibit clinical signs consistent with MVD. Throughout the study, these animals exhibited minimal abnormalities in body temperature, body weight, or clinical chemistry parameters. They were normal at gross examination, and no significant histopathologic lesions were observed.

**Figure 8:**
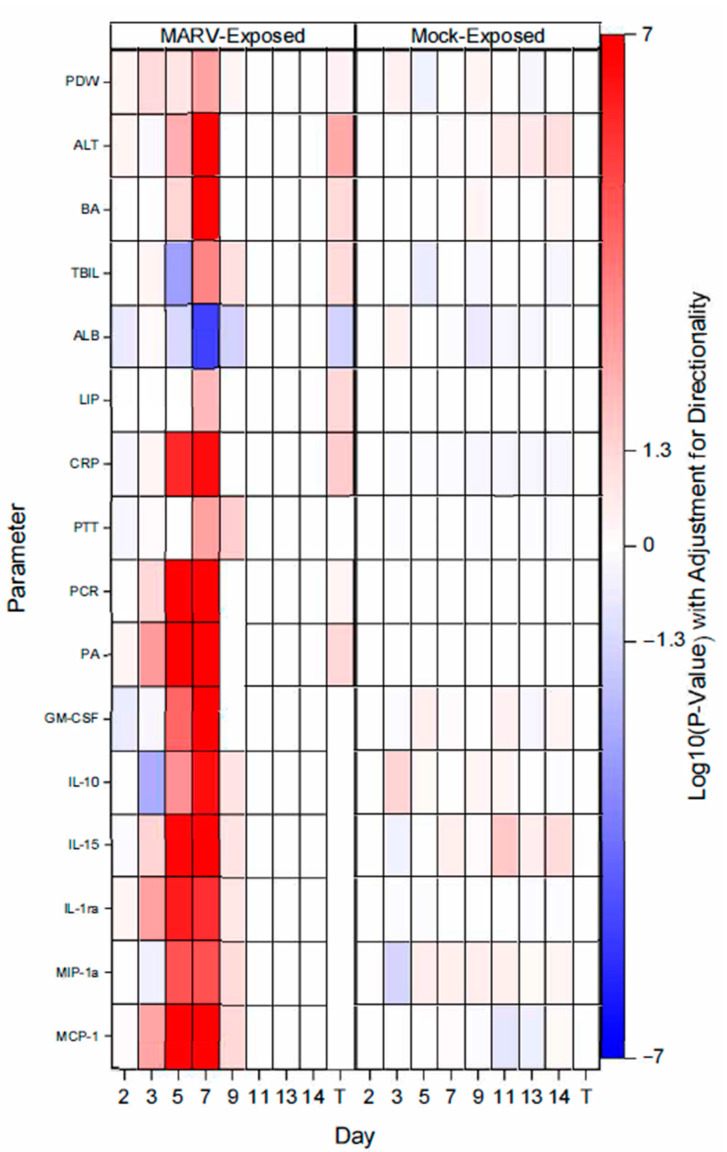
Onset of Abnormality Heat Map. PDW—platelet Distribution Width; ALT—alanine transaminase; BA—bile acids; TBIL—total bilirubin; ALB—albumin; LIP—lipemia; CRP—C-reactive protein; PTT—prothrombin time; PCR—viral RNA; PA—infectious virus via plaque assay; Terminal (T)—Terminal data collected from animals that met euthanasia criteria (unscheduled euthanasia) were combined and reported as a single time point.

For MARV-exposed animals, the earliest signs of disease appeared between Days 2 to 5 and included increased CRP levels, viral RNA and infectious virus, certain cytokines and sinus histiocytosis in the lymph nodes. Figure 8 displays the parameters that exhibited statistical significance for the onset of abnormality.

On Day 3, 63% (n = 10) had detectable levels of infectious viremia, and 31% (n = 5) exhibited low levels of viral RNA in the serum. Select tissues from animals euthanized on Day 2 contained detectable levels of viral RNA. No other biomarkers were identified at this early time point post exposure.

After Days 3 to 5 post exposure, many interleukins, which may be involved in inflammation, coagulopathy, and vascular permeability, were increased. Humans infected with filoviruses have shown high levels of pro-inflammatory cytokines (e.g., IL-1β, IL-4, IL-1RA, IL-6, IL-8, IL-15, and IL-16) and chemokines (e.g., MIP-1α, MIP-1β, MCP-1) [21,23,24].

On Days 5 and 6 post exposure, signs of reduced responsiveness, petechia, and increased rectal temperature were observed. As the study progressed, the remaining animals scored for: reduced intake in feed and/or enrichment and/or fluid and reduced (or lack of) stool output; and reduced responsiveness, petechia, and increased rectal temperatures were common. All serum samples for which data were available and determined from MARV-exposed animals beginning on Day 5 contained detectable levels of viral RNA and infectious virus.

Changes in clinical chemistry parameters consistent with disease became evident between Days 5 to 7. The hematology parameters correlated with disease were decreased lymphocytes and monocytes, increased granulocytes, and decreased platelets. Clinical chemistry parameters were indicative of liver malfunction—such as increases in ALP, ALT, GGT, BA, TBIL, and decreased ALB. The coagulation data suggest clotting times may have been increased in the latter stages of infection for some MARV-exposed animals. The most demonstrable changes in blood parameters and liver enzymes were evident by Day 7. Changes in chemokines and cytokines were also more frequent and more drastic in samples from infected animals euthanized later during the study.

Macroscopic observations attributed to MARV were first observed on Day 5 in one animal and in all infected animals at later time points. These included: discolored skin or challenge site; enlargement and/or discoloration of the axillary lymph nodes, spleen, liver, and lungs; changes in texture of spleen and/or liver (friability); and soft/green contents in large intestines. Discoloration was also found in the urinary bladder or in testicular parenchyma. Histopathology showed sinus histiocytosis, depletion of lymphocytes in the spleen and lymph nodes. The appearance of hepatocellular single cell necrosis (observed after Day 3), inflammatory reactions at the challenge site, and fibrin deposition in the spleen were also observed.

In summary, this study sought to define the disease course of MARV in cynomolgus macaques to determine if this model can serve as an acceptable means for the evaluation of medical countermeasures against MARV. The scheduled euthanasia provided the opportunity to study the earliest stages of the disease to determine if the measured biomarkers correlate with symptoms and the pathological progression of disease. The emphasis on the early stages of the disease also provided an opportunity to identify triggers that are appropriate for intervention in a therapeutic model. Viral load was the earliest reliable indicator of MARV infection. In addition, there were other biomarkers that reliably correlated with MARV-induced disease—based on comparing the data from the MARV-exposed to the mock-exposed animals—generally on and after Day 5. These biomarkers included: elevated total clinical scores; temperature changes (increased body temperature via telemetry was consistently detected on Day 4, increased rectal temperature was detected on Day 5); elevated ALT, ALP, BA, TBIL, CRP and decreased ALB values; decreased lymphocytes and platelets; and prolonged PTT.

## 5. Conclusions

The results of this study support the observation that MARV infection in cynomolgus macaques is a rapid systemic disease similar to the infection in humans under a compressed timescale. This study provides evidence for the application of this model to evaluate potential countermeasures against MARV. The model is severe, rapid, and exhibits characteristics amenable for both prophylactic and therapeutic testing. The experience from this study will be valuable in improving existing models to provide a reproducible testing platform, including the identification of appropriate biomarkers, the expected time for medical intervention, and the refinement of euthanasia criteria to allow reduction in the number of animals found dead in cage for optimal application of the model.

## Figures and Tables

**Table 1:** Animal group description, day of death, and final clinical score summary.

Animal ID	Sex	Group	Group Description	Day of Death	Found Dead or Euthanized	Final Clinical Score Prior to Euthanasia
594	F	B	MARV SE	Day 2 (S)	Euthanized	0
597	M	B	MARV SE	Day 2 (S)	Euthanized	0
585	F	B	MARV SE	Day 3 (S)	Euthanized	4
605	M	B	MARV SE	Day 3 (S)	Euthanized	1
592	F	B	MARV SE	Day 5 (S)	Euthanized	1
602	M	B	MARV SE	Day 5 (S)	Euthanized	2
589	F	B	MARV SE	Day 7 (S)	Euthanized	4
603	M	B	MARV SE	Day 7 (S)	Euthanized	4
584	F	B	MARV SE	Day 9 (S)	Euthanized	25
595	M	B	MARV SE	Day 9 ^¶^	FDIC ^¶^	N/A
591	F	C	MARV SP-TK	Day 7 (US)	Euthanized	33
588	F	C	MARV SP-TK	Day 8 (US)	Euthanized	16
593	F	C	MARV SP-TK	Day 8 (US)	Euthanized	20
596	M	C	MARV SP-TK	Day 8 (US)	Euthanized	27
601	M	D	MARV SP-TK	Day 8 (US)	FDIC	N/A
604	M	D	MARV SP-TK	Day 8 (US)	FDIC	N/A
587	F	C	MARV SP-TK	Day 9 (US)	FDIC	N/A
600	M	C	MARV SP-TK	Day 9 (US)	FDIC	N/A
590	F	A	SP-TK, Control	Day 14 (S)	Euthanized	1
598	M	A	SP-TK, Control	Day 14 (S)	Euthanized	0

F—Female; M—Male; MARV—Marburg virus; SE—Scheduled Euthanasia; SP-TK—Survival and Physiology Time Kinetics; S—Scheduled; US—Unscheduled; FDIC—found dead in cage. ^¶^ Animal was scheduled for euthanasia on Day 9 but was FDIC.

**Table 2:** Viral load in serum as determined by plaque assay (plaque-forming units/mL).

Animal ID	Group Description	Day of Death	Day 0	Day 2	Day 3	Day 5	Day 7	Day 9	Day 11	Day 13	Day 14	Terminal *
594	SE	Day 2 (S)	BDL	BDL	N/A	N/A	N/A	N/A	N/A	N/A	N/A	-
597	SE	Day 2 (S)	BDL	1.00 × 10^1^	N/A	N/A	N/A	N/A	N/A	N/A	N/A	-
585	SE	Day 3 (S)	BDL	N/A	BDL	N/A	N/A	N/A	N/A	N/A	N/A	-
605	SE	Day 3 (S)	BDL	N/A	6.13 × 10^2^	N/A	N/A	N/A	N/A	N/A	N/A	-
592	SE	Day 5 (S)	BDL	N/A	7.50 × 10^0^	5.50 × 10^3^	N/A	N/A	N/A	N/A	N/A	-
602	SE	Day 5 (S)	BDL	N/A	1.25 × 10^1^	1.33 × 10^6^	N/A	N/A	N/A	N/A	N/A	-
589	SE	Day 7 (S)	BDL	N/A	3.75 × 10^1^	1.08 × 10^6^	3.40 × 10^7^	N/A	N/A	N/A	N/A	-
603	SE	Day 7 (S)	BDL	N/A	BDL	2.00 × 10^4^	7.75 × 10^7^	N/A	N/A	N/A	N/A	-
584	SE	Day 9 (S)	BDL	N/A	BDL	ND	1.69 × 10^6^	7.50 × 10^7^	N/A	N/A	N/A	-
595	SE	Day 9 ^¶^	BDL	N/A	BDL	2.75 × 10^3^	1.06 × 10^8^	N/A	N/A	N/A	N/A	FDIC
591	SP-TK	Day 7 (US)	BDL	N/A	2.50 × 10^1^	7.13 × 10^5^	1.31 × 10^8^	N/A	N/A	N/A	N/A	ND
588	SP-TK	Day 8 (US)	BDL	N/A	1.15 × 10^2^	2.03 × 10^5^	2.43 × 10^7^	N/A	N/A	N/A	N/A	2.70 × 10^7^
593	SP-TK	Day 8 (US)	BDL	N/A	UD	1.26 × 10^6^	3.00 × 10^8^	N/A	N/A	N/A	N/A	3.09 × 10^8^
596	SP-TK	Day 8 (US)	BDL	N/A	1.00 × 10^1^	1.03 × 10^6^	1.55 × 10^8^	N/A	N/A	N/A	N/A	ND
601	SP-TK	Day 8 (US)	BDL	N/A	1.80 × 10^2^	1.95 × 10^7^	3.45 × 10^7^	N/A	N/A	N/A	N/A	FDIC
604	SP-TK	Day 8 (US)	BDL	N/A	1.50 × 10^1^	2.43 × 10^7^	8.88 × 10^7^	N/A	N/A	N/A	N/A	FDIC
587	SP-TK	Day 9 (US)	BDL	N/A	9.00 × 10^1^	1.38 × 10^5^	3.54 × 10^7^	FDIC	N/A	N/A	N/A	FDIC
600	SP-TK	Day 9 (US)	BDL	N/A	BDL	1.10 × 10^5^	3.24 × 10^7^	FDIC	N/A	N/A	N/A	FDIC

SP-TK—Survival and Clinical Pathology Time Kinetics; SE—Serial Sample and Euthanasia; BDL—below detection limit; UD—titer undetermined after repeat; FDIC—found dead in cage; S—Scheduled; US—Unscheduled; ^¶^ Animal was scheduled for euthanasia on Day 9 but was FDIC; ND—no data, sample not available; * Data from unscheduled moribund euthanasia occurring outside a usual collection time point.

**Table 3:** Viral load in gastrointestinal tract tissues as determined by plaque assay (plaque-forming units/gram of tissue).

Animal ID	Group Description	Day of Death	Colon	Rectum	Duodenum	Jejunum	Ileum	Stomach
594	SE	Day 2 (S)	BDL	BDL	BDL	BDL	BDL	BDL
597	SE	Day 2 (S)	BDL	BDL	BDL	BDL	BDL	BDL
585	SE	Day 3 (S)	BDL	BDL	UD	BDL	BDL	BDL
605	SE	Day 3 (S)	BDL	BDL	BDL	BDL	BDL	BDL
592	SE	Day 5 (S)	3.79 × 10^4^	4.55 × 10^4^	4.78 × 10^4^	BDL	BDL	BDL
602	SE	Day 5 (S)	5.00 × 10^5^	3.36 × 10^5^	4.02 × 10^5^	7.82 × 10^5^	7.54 × 10^4^	4.92 × 10^5^
589	SE	Day 7 (S)	2.48 × 10^7^	2.25 × 10^7^	2.01 × 10^7^	1.44 × 10^7^	2.10 × 10^7^	4.75 × 10^6^
603	SE	Day 7 (S)	5.28 × 10^7^	1.17 × 10^7^	1.38 × 10^7^	1.91 × 10^7^	1.79 × 10^7^	1.17 × 10^7^
584	SE	Day 9 (S)	1.28 × 10^8^	6.78 × 10^6^	3.18 × 10^7^	3.82 × 10^7^	4.96 × 10^7^	7.73 × 10^6^
595	SE	Day 9 ^¶^	1.33 × 10^8^	1.07 × 10^7^	2.50 × 10^7^	1.48 × 10^8^	1.66 × 10^8^	1.62 × 10^7^
591	SP-TK	Day 7 (US)	8.31 × 10^7^	5.66 × 10^7^	4.19 × 10^7^	6.82 × 10^7^	7.51 × 10^7^	9.97 × 10^6^
588	SP-TK	Day 8 (US)	2.43 × 10^8^	8.60 × 10^7^	UD	5.93 × 10^8^	2.97 × 10^8^	1.66 × 10^7^
593	SP-TK	Day 8 (US)	2.86 × 10^8^	6.54 × 10^7^	1.03 × 10^8^	9.68 × 10^7^	2.96 × 10^8^	7.44 × 10^7^
596	SP-TK	Day 8 (US)	1.28 × 10^8^	1.57 × 10^8^	4.66 × 10^7^	3.30 × 10^8^	1.98 × 10^8^	3.36 × 10^7^
601	SP-TK	Day 8 (US)	8.03 × 10^6^	3.36 × 10^8^	4.41 × 10^8^	2.24 × 10^7^	1.49 × 10^9^	1.90 × 10^7^
604	SP-TK	Day 8 (US)	3.01 × 10^7^	5.79 × 10^7^	6.12 × 10^7^	1.03 × 10^8^	8.36 × 10^7^	2.96 × 10^5^
587	SP-TK	Day 9 (US)	1.24 × 10^8^	3.63 × 10^7^	5.73 × 10^6^	1.41 × 10^8^	2.44 × 10^8^	8.21 × 10^6^
600	SP-TK	Day 9 (US)	9.04 × 10^7^	1.66 × 10^7^	6.20 × 10^7^	1.31 × 10^8^	1.55 × 10^8^	2.31 × 10^5^

SP-TK—Survival and Clinical Pathology Time Kinetics; SE—Serial Sample and Euthanasia; BDL—below detection limit; UD—titer undetermined after repeat; LN—lymph node; S—Scheduled; US—Unscheduled; ^¶^ Animal was scheduled for euthanasia on Day 9 but was FDIC.

**Table 4:** Viral load in liver and spleen as determined by plaque assay (plaque-forming units/gram of tissue).

Animal ID	Group Description	Day of Death	Posterior Medial Spleen	Anterior Spleen	Left Lateral Liver	Right Medial Liver
594	SE	Day 2 (S)	BDL	BDL	BDL	UD
597	SE	Day 2 (S)	1.57 × 10^2^	2.08 × 10^2^	BDL	BDL
585	SE	Day 3 (S)	UD	BDL	BDL	2.78 × 10^2^
605	SE	Day 3 (S)	1.18 × 10^6^	1.44 × 10^6^	8.55 × 10^5^	1.06 × 10^6^
592	SE	Day 5 (S)	1.95 × 10^6^	1.90 × 10^6^	1.06 × 10^7^	1.41 × 10^7^
602	SE	Day 5 (S)	5.33 × 10^7^	5.66 × 10^7^	2.33 × 10^8^	4.14 × 10^8^
589	SE	Day 7 (S)	4.13 × 10^8^	5.79 × 10^6^	1.25 × 10^9^	1.17 × 10^9^
603	SE	Day 7 (S)	5.92 × 10^8^	6.11 × 10^8^	5.95 × 10^8^	1.23 × 10^9^
584	SE	Day 9 (S)	4.51 × 10^8^	5.98 × 10^8^	8.48 × 10^8^	4.69 × 10^8^
595	SE	Day 9 ^¶^	6.68 × 10^8^	6.75 × 10^8^	6.09 × 10^8^	1.34 × 10^7^
591	SP-TK	Day 7 (US)	8.91 × 10^8^	1.11 × 10^9^	1.16 × 10^9^	1.14 × 10^8^
588	SP-TK	Day 8 (US)	1.01 × 10^9^	8.94 × 10^8^	1.51 × 10^9^	1.83 × 10^7^
593	SP-TK	Day 8 (US)	7.82 × 10^8^	1.40 × 10^9^	8.33 × 10^8^	1.29 × 10^8^
596	SP-TK	Day 8 (US)	8.75 × 10^8^	7.77 × 10^8^	2.69 × 10^9^	1.32 × 10^9^
601	SP-TK	Day 8 (US)	3.98 × 10^8^	4.62 × 10^8^	2.67 × 10^8^	8.01 × 10^7^
604	SP-TK	Day 8 (US)	4.64 × 10^7^	1.49 × 10^7^	8.29 × 10^7^	8.68 × 10^7^
587	SP-TK	Day 9 (US)	9.09 × 10^8^	1.06 × 10^9^	7.33 × 10^8^	1.11 × 10^9^
600	SP-TK	Day 9 (US)	3.63 × 10^8^	2.92 × 10^7^	6.20 × 10^8^	3.01 × 10^8^

SP-TK—Survival and Clinical Pathology Time Kinetics; SE—Serial Sample and Euthanasia; BDL—below detection limit; UD—titer undetermined after repeat; LN—lymph node; (S)—Scheduled; (US)—Unscheduled; ^¶^ Animal was scheduled for euthanasia on Day 9 but was FDIC.

**Table 5:** Viral load in tissues as determined by plaque assay (plaque-forming units/gram of tissue).

Animal ID	Group Description	Day of Death	Lung	Axillary LN	Adrenal Gland	Heart	Exposure Site	Right Inguinal LN	Mediastinal (Hilar) LN
594	SE	Day 2 (S)	BDL	1.39 × 10^2^	BDL	BDL	BDL	BDL	BDL
597	SE	Day 2 (S)	4.17 × 10^1^	2.30 × 10^3^	BDL	BDL	BDL	BDL	BDL
585	SE	Day 3 (S)	BDL	BDL	BDL	BDL	7.99 × 10^1^	BDL	BDL
605	SE	Day 3 (S)	1.50 × 10^2^	7.59 × 10^2^	3.33 × 10^2^	BDL	BDL	BDL	BDL
592	SE	Day 5 (S)	UD	1.12 × 10^8^	2.17 × 10^4^	BDL	BDL	BDL	BDL
602	SE	Day 5 (S)	3.13 × 10^5^	2.79 × 10^6^	6.70 × 10^5^	1.54 × 10^5^	1.45 × 10^5^	BDL	3.12 × 10^6^
589	SE	Day 7 (S)	7.10 × 10^5^	5.39 × 10^7^	4.17 × 10^7^	6.49 × 10^6^	3.25 × 10^5^	1.56 × 10^8^	7.03 × 10^7^
603	SE	Day 7 (S)	1.41 × 10^7^	8.43 × 10^6^	5.06 × 10^7^	1.34 × 10^7^	4.10 × 10^6^	3.55 × 10^7^	9.97 × 10^6^
584	SE	Day 9 (S)	3.86 × 10^7^	8.19 × 10^6^	5.21 × 10^7^	9.36 × 10^5^	6.24 × 10^5^	1.26 × 10^7^	3.33 × 10^7^
595	SE	Day 9 ^¶^	3.55 × 10^7^	1.31 × 10^8^	8.11 × 10^7^	3.51 × 10^7^	3.90 × 10^6^	5.71 × 10^7^	1.92 × 10^8^
591	SP-TK	Day 7 (US)	5.77 × 10^6^	9.02 × 10^7^	7.59 × 10^7^	9.76 × 10^6^	9.70 × 10^5^	1.12 × 10^8^	6.17 × 10^6^
588	SP-TK	Day 8 (US)	4.26 × 10^7^	1.84 × 10^8^	8.42 × 10^7^	2.85 × 10^6^	1.51 × 10^7^	2.56 × 10^7^	6.60 × 10^7^
593	SP-TK	Day 8 (US)	1.42 × 10^8^	2.98 × 10^8^	3.56 × 10^8^	3.32 × 10^7^	6.16 × 10^6^	3.08 × 10^7^	3.15 × 10^8^
596	SP-TK	Day 8 (US)	7.45 × 10^6^	6.08 × 10^7^	1.04 × 10^8^	6.80 × 10^6^	1.70 × 10^6^	1.12 × 10^8^	3.24 × 10^7^
601	SP-TK	Day 8 (US)	3.35 × 10^8^	3.47 × 10^6^	5.23 × 10^7^	2.89 × 10^7^	1.41 × 10^7^	1.02 × 10^8^	1.42 × 10^8^
604	SP-TK	Day 8 (US)	1.72 × 10^7^	1.44 × 10^7^	2.79 × 10^6^	6.48 × 10^6^	2.16 × 10^6^	2.89 × 10^8^	5.41 × 10^7^
587	SP-TK	Day 9 (US)	5.94 × 10^7^	1.46 × 10^8^	1.22 × 10^8^	1.50 × 10^7^	6.57 × 10^6^	7.95 × 10^7^	4.05 × 10^7^
600	SP-TK	Day 9 (US)	1.16 × 10^6^	2.28 × 10^7^	2.53 × 10^7^	6.15 × 10^5^	1.87 × 10^6^	2.24 × 10^7^	8.08 × 10^7^

SP-TK—Survival and Clinical Pathology Time Kinetics; SE—Serial Sample and Euthanasia; BDL—below detection limit; UD—titer undetermined after repeat; LN—lymph node; S—Scheduled; US—Unscheduled; ^¶^ Animal was scheduled for euthanasia on Day 9 but was FDIC.

## Data Availability

The data supporting the findings of this study are available within the article and its Supplementary Materials.

## Supplementary Materials

The following are available online at https://www.mdpi.com/article/10.3390/vaccines10081314/s1, Table S1. Viral Load in Serum as Determined by qRT-PCR Assay (GE/mL). Table S2. Viral Load in Gastrointestinal Tissues and Lymph Nodes as Determined by qRT-PCR Assay (GE/μg RNA). Table S3. Viral Load in Tissues as Determined by qRT-PCR Assay (GE/μg RNA). Figure S1. Mean with 95 percent confidence intervals over the course of the study for selected cytokines. Confidence interval was not plotted for sample size less than 3. For IFN- γ, IL-10, IL-15, IL-1ra, and IL-6, values were log-transformed and the geometric mean is displayed. Terminal (T) – Terminal data collected from animals that met euthanasia criteria (unscheduled euthanasia) were combined and reported as a single time point. (a) IFN- γ; (b) IL-1β; (c) IL-1ra; (d) IL-6; (e) IL-10; (f) IL-15. Figure S2. Geometric Mean with 95 percent confidence intervals over the course of the study for selected viral load. Confidence interval was not plotted for sample size less than 3. Study Day 0 serum results were below detection limit for all animals (and for mock exposed animals throughout) and were set to 2.5 pfu/mL for calculations and graphing. Tissue viral loads below limit of detection were set to 12.5 pfu/g for calculations and graphing. (a) Serum [PFU/ml]; (b) Axillary lymph node [PFU/g]; (c) Anterior spleen [PFU/g]; (d) Posterior medial spleen [PFU/g]; (e) Right medial liver [PFU/g]; (f) Left lateral liver [PFU/g]. Figure S3. Axillary lymph node. A. Day 14 PC PBS control, No. 590. Essentially normal tissue. 20x; B. Day 2 PC scheduled necropsy, No. 594. Essentially normal tissue. 20x; C. Day 3 PC scheduled necropsy, No. 585. Sinus histiocytosis. 20×; D. Day 7 PC scheduled necropsy, No. 603. Marked loss of lymphocytes, with lymphocytolysis, necrosis, hemorrhage, inflammation, fibrin and thrombosis. 10x; E. Day 8 unscheduled necropsy, No. 596. Severe lymphoid depletion with necrosis, inflammation, fibrin, hemorrhage and necrosis. 10x; F. Day 9 PC unscheduled necropsy, No. 584. Marked cortical lymphoid depletion with lymphocytolysis, thrombosis and paracortical hyperplasia. Note large immunoblast population. 40×. Figure S4. Spleen. A. Day 14 PC PBS control, No. 590. Essentially normal tissue. 20×; B. Day 2 PC scheduled necropsy, No. 594. Essentially normal tissue. 20×. C. Day 7 scheduled necropsy, No. 603. Moderate lymphoid depletion with lymphocytolysis and fibrin deposition. 20×; D. Day 8 PC unscheduled necropsy, No. 596. Marked lymphoid depletion with lymphocytolysis, marginal sinus congestion/hemorrhage and fibrin deposition. 20×; E. Day 9 PC unscheduled necropsy, No. 584. Marked lymphoid depletion with lymphocytolysis, fibrin and marginal sinus congestion/hemorrhage. 10x; F. Day 9 PC unscheduled necropsy, No. 584. Higher magnification of (E). 40×. Figure S5. Liver. A. Day 14 PC PBS control, No. 590. Minimal hepatocellular vacuolation consistent with increased glycogen. 40×; B. Day 2 PC scheduled necropsy, No. 594. Essentially normal tissue. 40×; C. Day 5 PC scheduled necropsy, No. 602. Single cell hepatocellular necrosis. 40×; D. Day 7 PC scheduled necropsy, No. 603. Minimal hepatocellular necrosis. 40×. E. Day 8 PC unscheduled necropsy, No. 596. Moderate single cell hepatocellular necrosis with inflammation. 40×; F. Day 9 unscheduled necropsy, No. 584. Marked hepaticellular necrosis with inflammation and fibrin. 40×.
