# Supplementary material for: Development of a Well-Characterized Cynomolgus Macaque Model of Marburg Virus Disease for Support of Vaccine and Therapy Development
Source: Vaccines (Basel). 2022 Aug 14;10(8):1314. doi: 10.3390/vaccines10081314 (PMC9414819; doi:10.3390/vaccines10081314)
Supplement: Supplementary file 1 [file vaccines-10-01314-s001.zip › supplementary Figures.pdf]

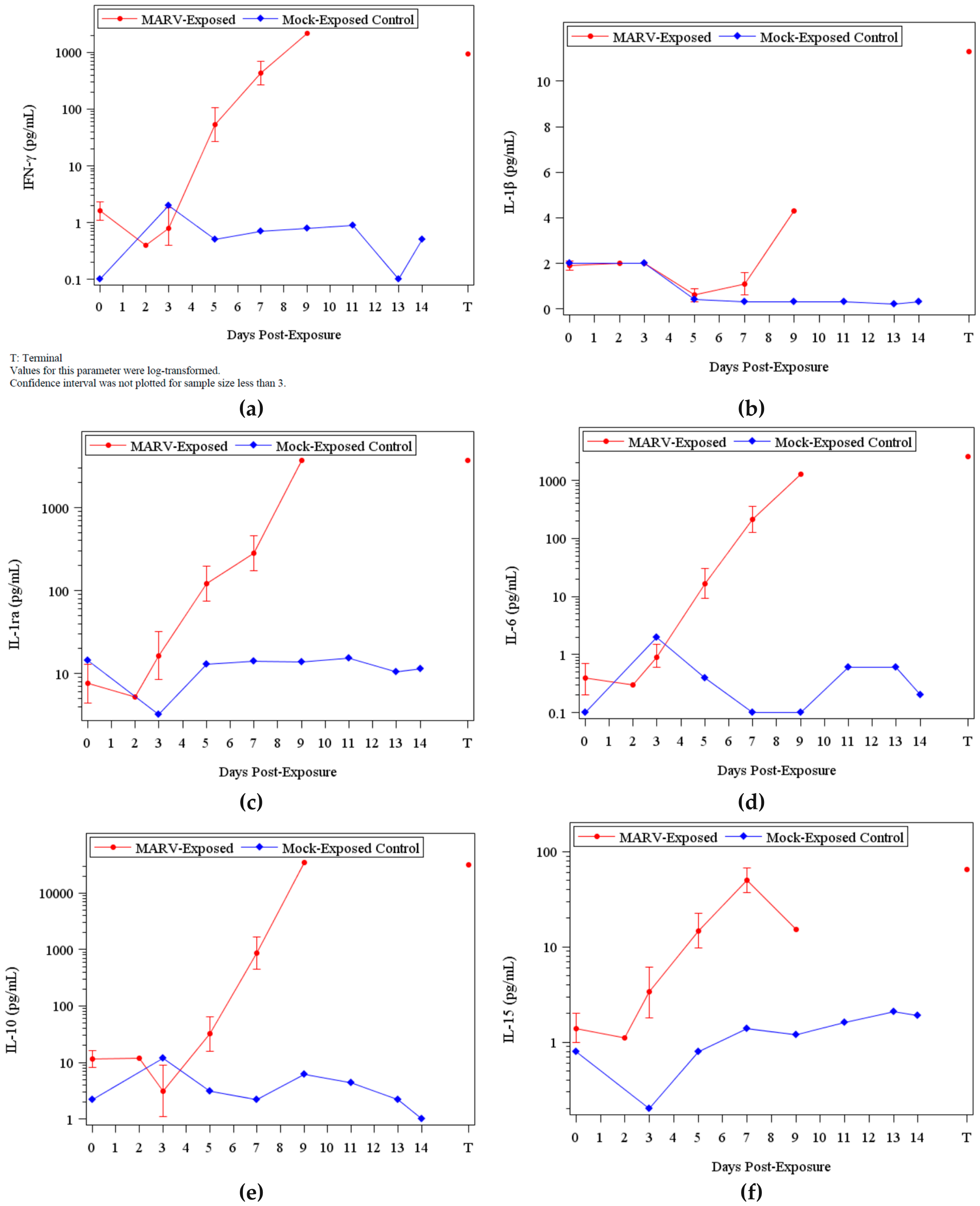

Figure S1. Mean with 95 percent confidence intervals over the course of the study for selected cytokines. Confidence interval was not plotted for sample size less than 3. For IFN- γ, IL-10, IL-15, IL-1ra, and IL-6, values were log-transformed and the geometric mean is displayed. Terminal (T) – Terminal data collected from animals that met euthanasia criteria (unscheduled euthanasia) were combined and reported as a single time point. (**a**) IFN- γ; (**b**) IL-1β; (**c**) IL-1ra; (**d**) IL-6; (**e**) IL-10; (**f**) IL-15.

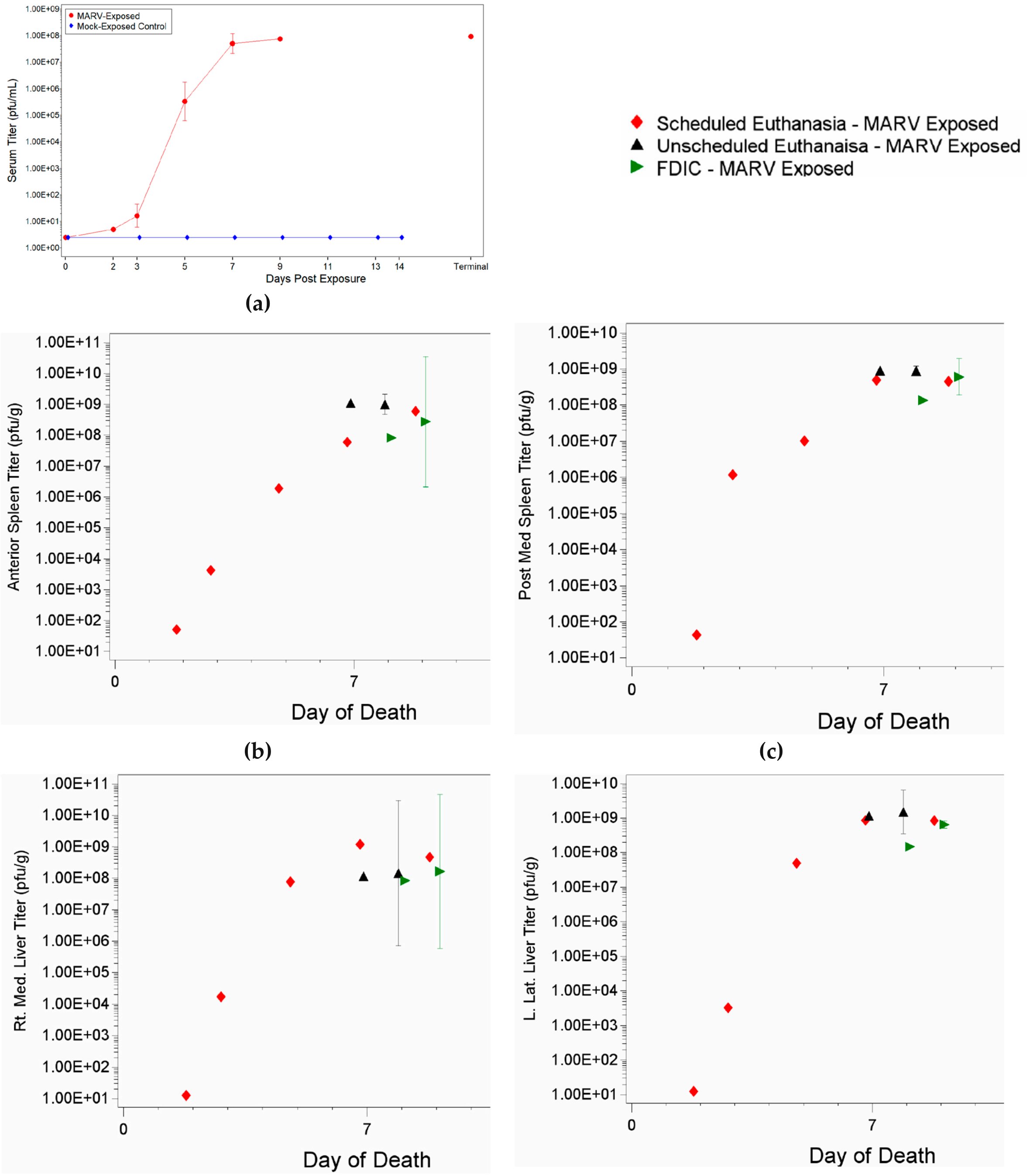

Figure S2. Geometric Mean with 95 percent confidence intervals over the course of the study for selected viral load. Confidence interval was not plotted for sample size less than 3. Study Day 0 serum results were below detection limit for all animals (and for mock exposed animals throughout) and were set to 2.5 pfu/mL for calculations and graphing. Tissue viral loads below limit of detection were set to 12.5 pfu/g for calculations and graphing. (**a**) Serum [PFU/ml]; (**b**) Axillary lymph node [PFU/g]; (**c**) Anterior spleen [PFU/g]; (**d**) Posterior medial spleen [PFU/g]; (**e**) Right medial liver [PFU/g]; (**f**) Left lateral liver [PFU/g].

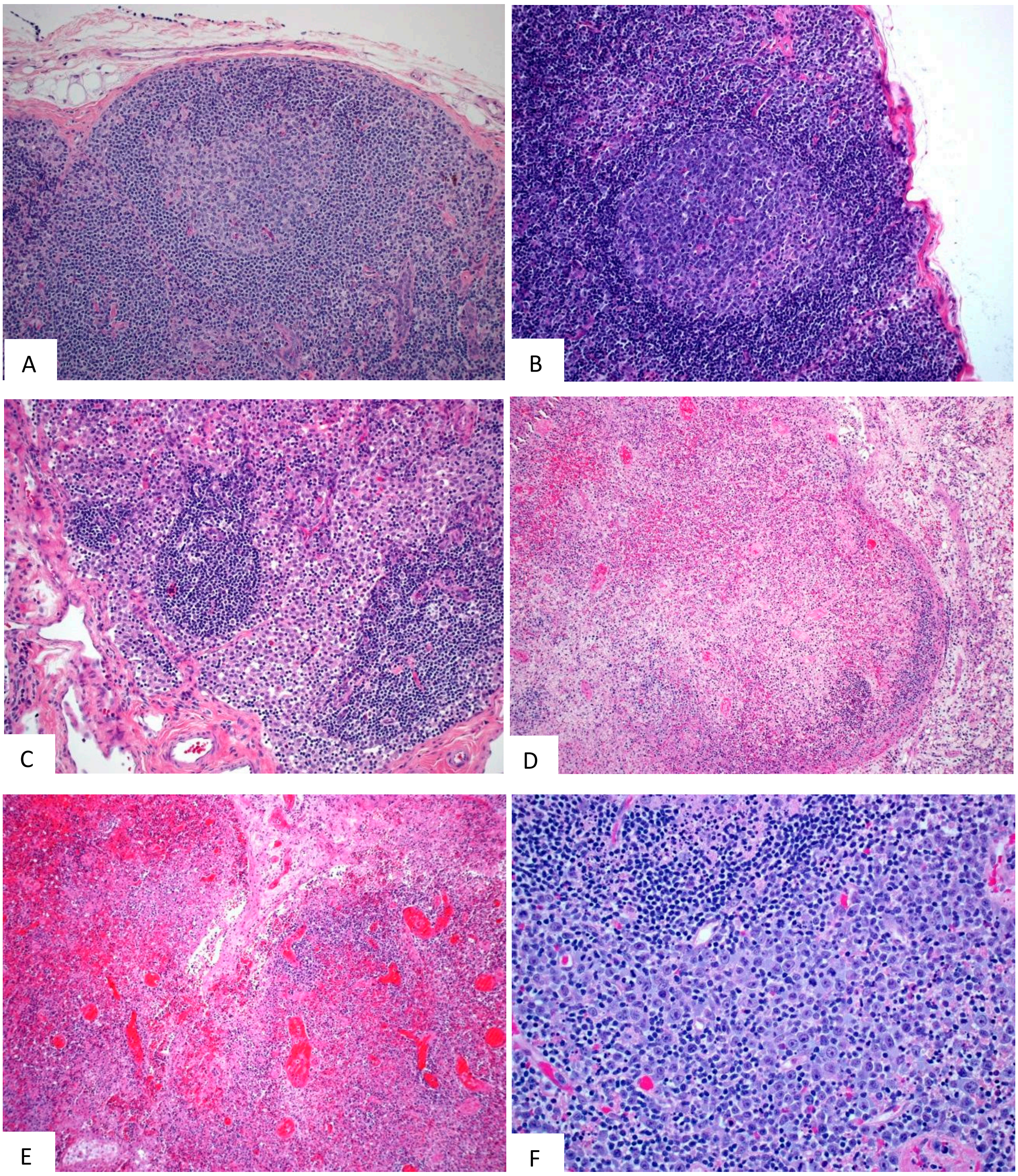

Figure S3. Axillary lymph node. A. Day 14 PC PBS control, No. 590. Essentially normal tissue. 20x; B. Day 2 PC scheduled necropsy, No. 594. Essentially normal tissue. 20x; C. Day 3 PC scheduled necropsy, No. 585. Sinus histiocytosis. 20x; D. Day 7 PC scheduled necropsy, No. 603. Marked loss of lymphocytes, with lymphocytolysis, necrosis, hemorrhage, inflammation, fibrin and thrombosis. 10x; E. Day 8 unscheduled necropsy, No. 596. Severe lymphoid depletion with necrosis, inflammation, fibrin, hemorrhage and necrosis. 10x; F. Day 9 PC unscheduled necropsy, No. 584. Marked cortical lymphoid depletion with lymphocytolysis, thrombosis and paracortical hyperplasia. Note large immunoblast population. 40x.

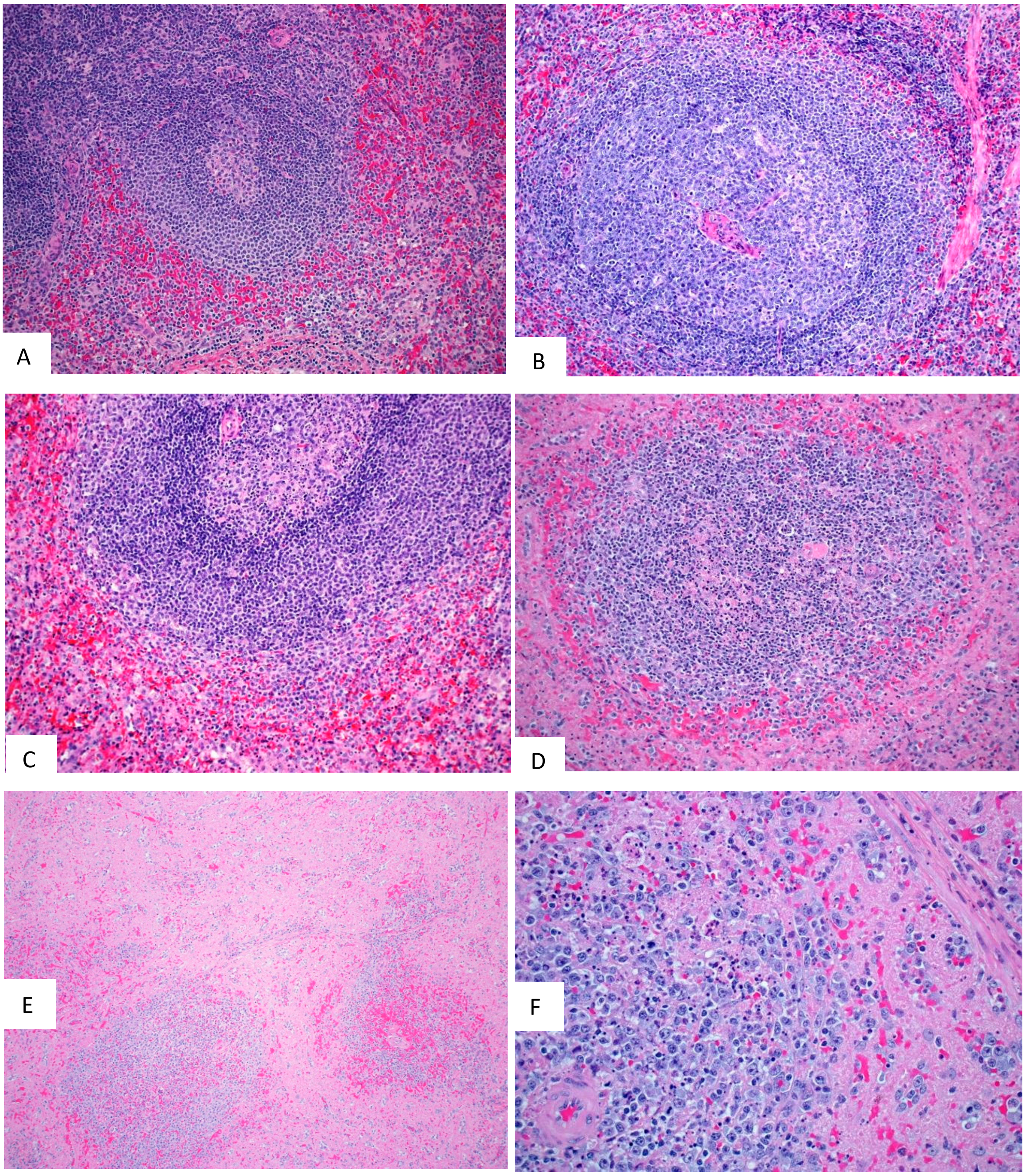

Figure S4. Spleen. A. Day 14 PC PBS control, No. 590. Essentially normal tissue. 20x; B. Day 2 PC scheduled necropsy, No. 594. Essentially normal tissue. 20x. C. Day 7 scheduled necropsy, No. 603. Moderate lymphoid depletion with lymphocytolysis and fibrin deposition. 20x; D. Day 8 PC unscheduled necropsy, No. 596. Marked lymphoid depletion with lymphocytolysis, marginal sinus congestion/hemorrhage and fibrin deposition. 20x; E. Day 9 PC unscheduled necropsy, No. 584. Marked lymphoid depletion with lymphocytolysis, fibrin and marginal sinus congestion/hemorrhage. 10x; F. Day 9 PC unscheduled necropsy, No. 584. Higher magnification of (E). 40x.

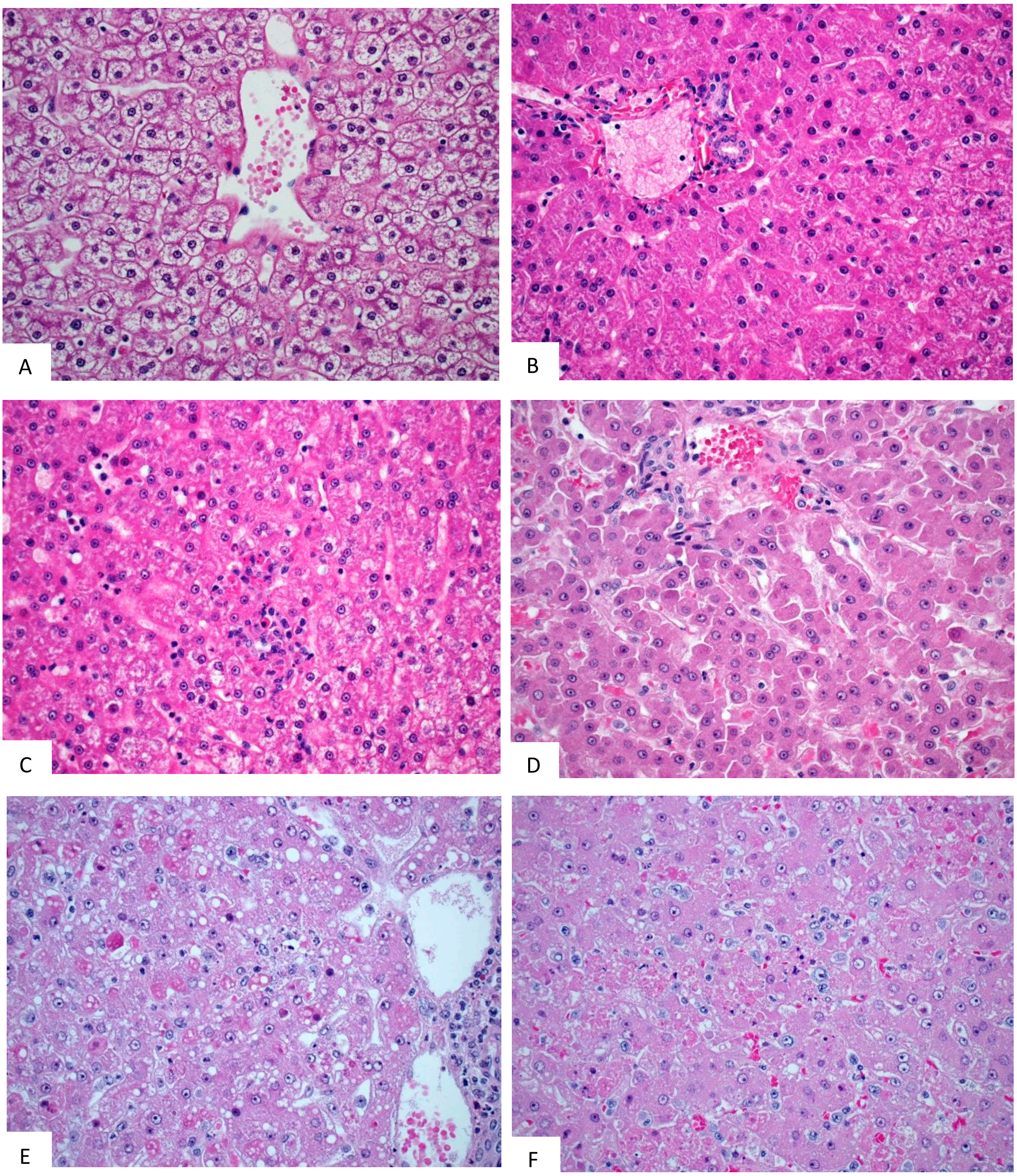

Figure S5. Liver. A. Day 14 PC PBS control, No. 590. Minimal hepatocellular vacuolation consistent with increased glycogen. 40x; B. Day 2 PC scheduled necropsy, No. 594. Essentially normal tissue. 40x; C. Day 5 PC scheduled necropsy, No. 602. Single cell hepatocellular necrosis. 40x; D. Day 7 PC scheduled necropsy, No. 603. Minimal hepatocellular necrosis. 40x. E. Day 8 PC unscheduled necropsy, No. 596. Moderate single cell hepatocellular necrosis with inflammation. 40x; F. Day 9 unscheduled necropsy, No. 584. Marked hepaticellular necrosis with inflammation and fibrin. 40x.
