# Supplementary material for: Development of a Well-Characterized Cynomolgus Macaque Model of Marburg Virus Disease for Support of Vaccine and Therapy Development
Source: Vaccines (Basel). 2022 Aug 14;10(8):1314. doi: 10.3390/vaccines10081314 (PMC9414819; doi:10.3390/vaccines10081314)
Supplement: Supplementary file 1 [file vaccines-10-01314-s001.zip › supplementary Tables.pdf]

Table S1. Viral Load in Serum as Determined by qRT-PCR Assay (GE/mL)

Animal ID	Group Description	Day of death	Day 0	Day 2	Day 3	Day 5	Day 7	Day 9	Day 11	Day 13	Day 14	Terminal
**594**	SE	Day 2 (S)	BDL	BDL	N/A	N/A	N/A	N/A	N/A	N/A	N/A	N/A
**597**	SE	Day 2 (S)	BDL	BDL	N/A	N/A	N/A	N/A	N/A	N/A	N/A	N/A
**585**	SE	Day 3 (S)	BDL	N/A	BDL	N/A	N/A	N/A	N/A	N/A	N/A	N/A
**605**	SE	Day 3 (S)	BDL	N/A	2.43×10^{4}	N/A	N/A	N/A	N/A	N/A	N/A	N/A
**592**	SE	Day 5 (S)	BDL	N/A	BDL	2.96×10^{5}	N/A	N/A	N/A	N/A	N/A	N/A
**602**	SE	Day 5 (S)	BDL	N/A	BDL	4.43×10^{7}	N/A	N/A	N/A	N/A	N/A	N/A
**589**	SE	Day 7 (S)	BDL	N/A	4.99×10^{2}	3.09×10^{7}	6.91×10^{8}	N/A	N/A	N/A	N/A	N/A
**603**	SE	Day 7 (S)	BDL	N/A	BDL	6.26×10^{5}	2.14×10^{10}	N/A	N/A	N/A	N/A	N/A
**584**	SE	Day 9 (S)	BDL	N/A	BDL	ND	2.45×10^{7}	5.62×10^{10}	N/A	N/A	N/A	N/A
**595**	SE	Day 9¶	BDL	N/A	BDL	4.29×10^{4}	6.31×10^{9}	N/A	N/A	N/A	N/A	FDIC
**591**	SP-TK	Day 7 (US)	BDL	N/A	BDL	2.96×10^{7}	1.47×10^{10}	N/A	N/A	N/A	N/A	ND
**588**	SP-TK	Day 8 (US)	BDL	N/A	3.09×10^{3}	5.50×10^{6}	3.45×10^{8}	N/A	N/A	N/A	N/A	4.64×10^{7}
**593**	SP-TK	Day 8 (US)	BDL	N/A	BDL	2.26×10^{7}	7.19×10^{10}	N/A	N/A	N/A	N/A	BDL
**596**	SP-TK	Day 8 (US)	BDL	N/A	BDL	2.10×10^{7}	2.14×10^{10}	N/A	N/A	N/A	N/A	ND
**601**	SP-TK	Day 8 (US)	BDL	N/A	1.15×10^{4}	1.04×10^{9}	2.58×10^{10}	N/A	N/A	N/A	N/A	FDIC
**604**	SP-TK	Day 8 (US)	BDL	N/A	BDL	7.43×10^{8}	1.15×10^{10}	N/A	N/A	N/A	N/A	FDIC
**587**	SP-TK	Day 9 (US)	BDL	N/A	3.42×10^{3}	2.67×10^{6}	1.42×10^{10}	FDIC	N/A	N/A	N/A	FDIC
**600**	SP-TK	Day 9 (US)	BDL	N/A	BDL	1.19×10^{6}	9.49×10^{8}	FDIC	N/A	N/A	N/A	FDIC

SP-TK—Survival and Clinical Pathology Time Kinetics; SE – Serial Sample and Euthanasia; BDL - below detection limit; LN – lymph node; S – Scheduled; US – Unscheduled; ND – no data, sufficient sample not available; ¶ Animal was scheduled for euthanasia on Day 9, but was FDIC.

Table S2. Viral Load in Gastrointestinal Tissues and Lymph Nodes as Determined by qRT-PCR Assay (GE/μg RNA)

Animal ID	Group Description	Day of death	Stomach	Duodenum	Jejunum	Ileum	Colon	Rectum	Axillary LN	Right Inguinal LN	Mediastinal LN
**594**	SE	Day 2 (S)	BDL	BDL	BDL	BDL	BDL	BDL	4.31 x 10^{0}	BDL	BDL
**597**	SE	Day 2 (S)	BDL	BDL	BDL	BDL	BDL	BDL	BDL	BDL	BDL
**585**	SE	Day 3 (S)	BDL	BDL	BDL	BDL	BDL	BDL	BDL	BDL	BDL
**605**	SE	Day 3 (S)	BDL	BDL	BDL	2.40 x 10^{2}	BDL	BDL	8.65 x 10^{2}	5.23 x 10^{3}	4.03 x 10^{3}
**592**	SE	Day 5 (S)	BDL	BDL	2.37 x 10^{1}	1.30 x 10^{2}	BDL	3.78 x 10^{2}	5.28 x 10^{6}	BDL	6.56 x 10^{1}
**602**	SE	Day 5 (S)	BDL	BDL	1.46 x 10^{4}	1.32 x 10^{4}	BDL	BDL	1.48 x 10^{5}	5.19 x 10^{5}	4.98 x 10^{5}
**589**	SE	Day 7 (S)	4.56 x 10^{1}	BDL	1.99 x 10^{5}	3.22 x 10^{5}	1.38 x 10^{4}	BDL	4.77 x 10^{6}	4.19 x 10^{6}	1.58 x 10^{6}
**603**	SE	Day 7 (S)	BDL	BDL	BDL	BDL	1.34 x 10^{3}	BDL	1.47 x 10^{6}	1.02 x 10^{6}	1.83 x 10^{5}
**584**	SE	Day 9 (S)	BDL	1.57 x 10^{4}	BDL	5.45 x 10^{5}	1.10 x 10^{4}	8.64 x 10^{3}	2.27 x 10^{5}	1.43 x 10^{6}	1.14 x 10^{4}
**595**	SE	Day 9¶	BDL	BDL	BDL	BDL	BDL	BDL	3.18 x 10^{6}	5.48 x 10^{5}	4.75 x 10^{6}
**591**	SP-TK	Day 7 (US)	8.13 x 10^{3}	BDL	4.05 x 10^{3}	4.82 x 10^{5}	3.82 x 10^{4}	5.27 x 10^{3}	2.41 x 10^{6}	9.56 x 10^{4}	9.06 x 10^{5}
**588**	SP-TK	Day 8 (US)	BDL	BDL	BDL	1.49 x 10^{3}	BDL	BDL	5.51 x 10^{6}	4.43 x 10^{6}	1.37 x 10^{7}
**593**	SP-TK	Day 8 (US)	BDL	2.80 x 10^{3}	3.06 x 10^{2}	5.41 x 10^{4}	BDL	BDL	1.46 x 10^{8}	5.07 x 10^{1}	1.22 x 10^{7}
**596**	SP-TK	Day 8 (US)	BDL	9.00 x 10^{3}	5.26 x 10^{5}	1.25 x 10^{5}	BDL	BDL	1.22 x 10^{8}	4.81 x 10^{6}	7.02 x 10^{6}
**601**	SP-TK	Day 8 (US)	BDL	2.24 x 10^{2}	BDL	1.87 x 10^{5}	3.16 x 10^{3}	5.29 x 10^{3}	4.43 x 10^{7}	2.87 x 10^{7}	4.11 x 10^{7}
**604**	SP-TK	Day 8 (US)	BDL	BDL	BDL	2.66 x 10^{4}	BDL	BDL	6.03 x 10^{7}	9.17 x 10^{6}	5.47 x 10^{7}
**587**	SP-TK	Day 9 (US)	BDL	BDL	BDL	4.21 x 10^{1}	BDL	BDL	2.93 x 10^{6}	2.38 x 10^{6}	7.74 x 10^{6}
**600**	SP-TK	Day 9 (US)	BDL	BDL	BDL	BDL	BDL	BDL	2.45 x 10^{3}	2.51 x 10^{6}	4.20 x 10^{7}

SP-TK—Survival and Clinical Pathology Time Kinetics; SE – Serial Sample and Euthanasia; BDL - below detection limit; LN – lymph node; S – Scheduled; US – Unscheduled; ¶ Animal was scheduled for euthanasia on Day 9, but was FDIC.

Table S3. Viral Load in Tissues as Determined by qRT-PCR Assay (GE/μg RNA)

Animal ID	Group Description	Day of death	Heart	Lung	Spleen	Liver	Challenge Site	Adrenal Gland
**594**	SE	Day 2 (S)	BDL	BDL	BDL	4.77×10^{0}	BDL	BDL
**597**	SE	Day 2 (S)	BDL	BDL	7.77×10^{2}	2.67×10^{1}	BDL	2.01×10^{2}
**585**	SE	Day 3 (S)	BDL	BDL	BDL	4.19×10^{1}	2.28×10^{1}	ND
**605**	SE	Day 3 (S)	6.57×10^{2}	BDL	1.89×10^{5}	7.18×10^{4}	2.86×10^{4}	1.00×10^{3}
**592**	SE	Day 5 (S)	6.91×10^{3}	BDL	3.83×10^{5}	5.62×10^{5}	4.52×10^{3}	9.26×10^{3}
**602**	SE	Day 5 (S)	1.10×10^{4}	BDL	3.69×10^{6}	4.92×10^{6}	2.70×10^{5}	2.61×10^{4}
**589**	SE	Day 7 (S)	2.21×10^{6}	BDL	8.63×10^{7}	2.32×10^{8}	9.91×10^{4}	5.52×10^{6}
**603**	SE	Day 7 (S)	1.67×10^{6}	BDL	1.37×10^{8}	3.31×10^{8}	3.11×10^{5}	6.37×10^{6}
**584**	SE	Day 9 (S)	5.00×10^{5}	9.39×10^{2}	2.03×10^{8}	2.79×10^{8}	3.72×10^{5}	3.38×10^{4}
**595**	SE	Day 9¶	2.46×10^{6}	BDL	2.79×10^{8}	1.81×10^{8}	4.19×10^{5}	7.03×10^{6}
**591**	SP-TK	Day 7 (US)	1.16×10^{6}	1.25×10^{4}	7.19×10^{7}	1.64×10^{8}	9.73×10^{5}	5.67×10^{3}
**588**	SP-TK	Day 8 (US)	1.25×10^{6}	BDL	1.02×10^{8}	2.69×10^{8}	1.51×10^{6}	2.96×10^{6}
**593**	SP-TK	Day 8 (US)	4.78×10^{6}	7.37×10^{4}	3.75×10^{8}	2.42×10^{8}	2.67×10^{6}	8.72×10^{6}
**596**	SP-TK	Day 8 (US)	2.25×10^{6}	BDL	1.98×10^{8}	1.62×10^{8}	3.25×10^{5}	4.05×10^{7}
**601**	SP-TK	Day 8 (US)	2.96×10^{6}	BDL	7.98×10^{7}	1.09×10^{8}	1.57×10^{6}	4.25×10^{7}
**604**	SP-TK	Day 8 (US)	2.66×10^{6}	BDL	2.50×10^{8}	3.05×10^{8}	9.79×10^{5}	6.26×10^{6}
**587**	SP-TK	Day 9 (US)	1.59×10^{6}	BDL	2.22×10^{8}	2.44×10^{8}	1.09×10^{5}	7.01×10^{6}
**600**	SP-TK	Day 9 (US)	3.03×10^{5}	BDL	1.65×10^{8}	2.25×10^{8}	BDL	3.89×10^{6}

SP-TK—Survival and Clinical Pathology Time Kinetics; SE – Serial Sample and Euthanasia; BDL - below detection limit; LN – lymph node; S – Scheduled; US – Unscheduled; ND – no data, sufficient sample not available; ¶ Animal was scheduled for euthanasia on Day 9, but was FDIC.
